# Horizontal inequity in self-reported morbidity and untreated morbidity in India: Evidence from National Sample Survey Data

**DOI:** 10.1186/s12939-020-01376-0

**Published:** 2021-01-28

**Authors:** Veenapani Rajeev Verma, Umakant Dash

**Affiliations:** Department of Humanities and Social Sciences, Indian Institute of Technology Madras, Chennai, India

**Keywords:** Horizontal inequities, Erreygers concentration index, Self-reported morbidity, Untreated morbidity, Decomposition of inequalities, National Sample Survey Data

## Abstract

**Background:**

Health outcomes in India are characterized by pervasive inequities due to deeply entrenched socio-economic gradients amongst the population. Therefore, it is imperative to investigate these systematic disparities in health, however, evidence of inequities does not commensurate with its policy objectives in India. Thus, our paper aims to examine the magnitude of and trends in horizontal inequities in self-reported morbidity and untreated morbidity in India over the period of 2004 to 2017–18.

**Methods:**

The study used cross-sectional data from nationwide healthcare surveys conducted in 2004, 2014 and 2017–18 encompassing sample size of 3,85,055; 3,35,499 and 5,57,887 individuals respectively. Erreygers concentration indices were employed to discern the magnitude and trend in horizontal inequities in self-reported morbidity and untreated morbidity. Need standardized concentration indices were further used to unravel the inter-regional and intra-regional income related inequities in outcomes of interest. Additionally, regression based decomposition approach was applied to ascertain the contributions of both legitimate and illegitimate factors in the measured inequalities.

**Results:**

Estimates were indicative of profound inequities in self-reported morbidity as inequity indices were positive and significant for all study years, connoting better-off reporting more morbidity, given their needs. These inequities however, declined marginally from 2004(HI: 0.049, *p*< 0.01) to 2017–18(HI: 0.045, *P*< 0.01). Untreated morbidity exhibited pro-poor inequities with negative concentration indices. Albeit, significant reduction in horizontal inequity was found from 2004(HI= − 0.103, *p*< 0.01) to 2017–18(HI = − 0.048, p< 0.01) in treatment seeking over the years. The largest contribution of inequality for both outcomes stemmed from illegitimate variables in all the study years. Our findings also elucidated inter-state heterogeneities in inequities with high-income states like Andhra Pradesh, Kerala and West Bengal evincing inequities greater than all India estimates and Northeastern states divulged equity in reporting morbidity. Inequities in untreated morbidity converged for most states except in Punjab, Chhattisgarh and Himachal Pradesh where widening of inequities were observed from 2004 to 2017–18.

**Conclusions:**

Pro-rich and pro-poor inequities in reported and untreated morbidities respectively persisted from 2004 to 2017–18 despite reforms in Indian healthcare. Magnitude of these inequities declined marginally over the years. Health policy in India should strive for targeted interventions closing inequity gap.

**Supplementary Information:**

The online version contains supplementary material available at 10.1186/s12939-020-01376-0.

## Introduction

Inequality in health is an empirical notion that refers to differences in health status between different groups. The term does not refer generically to just any inequalities between any population groups, but very specifically to disparities between groups of people categorized a priori according to some important features of their underlying social position [1]. It is a multi-dimensional concept, consisting of technical and normative judgements in the choice of appropriate metrics [2]. Health equity resonates with the Sustainable Development Goal’s overarching principle of leaving no one behind and the implicit moral imperative of social justice [3]. The WHO Commission on Social Determinants of Health further asserts health inequities as the differences that are systematic, avoidable and unfair. Such socio-economic inequities are ubiquitous in the health outcomes of developing world including India, where healthcare consumption is profoundly characterized by socio-economic gradient whereby, those who are socially and economically more disadvantaged have less access to and utilize less services. The pursuit of equity is deeply entrenched in the principles of Universal Health Coverage, which is also envisaged in the policies formulated in India. National Health Policy 2017 [4] directed that budgetary allocations would ensure horizontal equity through targeting specific population sub groups, geographical areas, health care services and gender related issues. However, social and economic inequality in healthcare remains an unprecedented challenge in India and as the nation commits to embark on the journey towards Universal Health Coverage, it becomes imperative to explore the dimension of equity to promulgate inclusive policies.

The distributive justice theory of Aristotle distinguishes between vertical and horizontal equity [5]. Horizontal equity enunciates the concept of equal treatment for equal *need*, irrespective of other socio-economic characteristics such as income, education, place of residence, social group etc. Whilst, vertical equity refers to unequal treatment for unequal needs. However, *need* is rather elusive and intractable concept that makes measurement of horizontal inequities more challenging than health inequalities, not only for their requirements for data on determinants of health but also for ethical considerations [6]. The degree to which the health inequality is inequitable is discerned via need-adjustment. Literature commonly identifies the *need* with ill-health suggesting that people with similar health statuses have same need and persons with dissimilar health statuses have different needs [7]. The need or legitimate variables are not amenable to the policy intervention and thus, considered as fair; whereas, health inequality due to non-need or illegitimate factors which are amenable to policy intervention is considered as unfair. Once healthcare outcome has been standardized by need, the resultant systematic disparities in health captures the degree to which the health inequality is inequitable.

Substantive income inequalities are prevalent in India, as indicated by high Gini index of 37.8 in 2011 (WHO). Inequality in India is not only characterized by higher estimates vis .a. vis countries at similar level of economic development, but has exhibited upward trend over time, especially since 1990’s [8]. The Gini index for consumption expenditure augmented from 0.30 to 0.37 from 1983 to 2011–12. Besides, the index value based on income level and wealth was colossally high at 0.54 and 0.75 in 2011-12. Further, the share of national income accruing to top 1% income earners was 22% in 2014 [9], which remained unvaried in 2018. As per the latest estimates, in 2018, top 1% captured 21.4% and top 10% garnered 56.1% of income in India [10]. Evidence on socio-economic inequalities in access and utilization of healthcare services is also ubiquitous in India, albeit the literature in Indian context is dominated by empirical studies delving into maternal and child health outcomes only [11–14]. The studies enquiring the degree to which health inequality is inequitable is further scarce with very few studies unraveling horizontal inequities in India [15–17]. Additionally, many healthcare reforms have been launched in India during last few years with concerted efforts to abridge inequality gaps making it imperative to evaluate the progress towards achieving the same, however, there is paucity of literature in this context. Also, there is a substantial variation in the health outcomes between the regions which needs to be examined. Analysis at a regional level is pertinent to discern the evidence informing how policies, programs and practices can be aligned to promote better health amongst the disadvantaged. Despite its policy relevance, current literature circumvents the subject of regional variations in most of the studies. Therefore, an analysis of extent, trends and determinants of horizontal inequities in the prevalence of morbidity and utilization of health services at regional level needs to be undertaken.

In the absence of alternative data sources and electronic records, most of the studies in Low and Middle Income Countries (LMIC) setting use self-reporting of morbidity as compared to the objective assessment to estimate socio-economic inequalities to gauge disease prevalence [18–21]. Self-reported health status as a method of assessing morbidity conditions of a given population has been demonstrated to be effective in capturing health variation in a population. The proponents of Self-reported health measures asserts that self-reported measures are a tool with desirable properties such as *Stability*, *Consistency* and *Good Test-Retest Reliability*. Self-rated health is a more inclusive and accurate measure of health-status as it encapsulates full array of illness debilitating a person and possess a realm of possibility to even capture symptoms of disease as yet undiagnosed prodromal/ prodromal stages. Furthermore, self-rating of health embodies complex human judgement about severity of current illness and provides a dynamic evaluation discerning both the trajectory and current level of health [22]. The seminal work by Idler and Benyamini, 1997 [23] reviewed a set of studies and found out that in great majority of cases; self-ratings adds something more to the prediction of mortality and concluded that self-ratings represent a source of valuable data on health status presenting an indispensable dimension of health status, without which individual health status cannot be assessed. There is a good basis for using self-rated health as an outcome as it can provide more holistic view of health which may not be reflected in objective measures such as those based on specific medical diagnosis [24]. Self-rated health influences behavior that subsequently affect health status. It is pertinent to investigate the inequities in self-reported morbidities as heterogeneities in perceptions subsequently, influences the health seeking behavior. It is desirable to examine a dynamic rather than static perspective on health as is subsumed in self-reported measures. Furthermore, objective measures such as consultation data has some constraints e.g. it does not reflect all the health problems in a population since many of those are not bought to attention of healthcare services [25]. Subjective measures such as self-reported health status on the other hand, is extremely valuable measure of health as it gauges what really matters and is an indicator of patient’s empowerment [26]. While assessing a person’s health condition and health-care demand, it is essential to take perception of individual about his/her health into consideration; hence, self-reported health status represents a summary statement about how numerous aspects of health, both subjective and objective are combined within perceptual framework of individual respondent [27]. Some evidence purported that self-reported health could also predict hospitalization and specialist consultation better than diagnosed health conditions [26]. From the sociological perspective, it is argued that self-reported illness represents well-being of an individual more than an objective, medically-confirmed disease 28]. Self-reports has been used profusely in developing countries using large-scale demographic and health surveys (DHS) for estimating prevalence of illnesses and remains one of the most widely used methods in clinical, public health, social and economic research [29]. Specifically, in a country like India, self-reported measure is both desirable and feasible as objective data on health is scarce whereas, self-reported measures are easy and inexpensive to collect and studies have also demonstrated them to be a good predictor of mortality and functionability, even after controlling for other objective health measures. Evidence from other low income setting of Bangladesh demonstrates both the multidimensional nature and effective predictive power of relatively simple and low-cost measure of self-reported health and establishes its validity and supports the notion that individuals can effectively assess their own health status even in settings of poor education and lower level of interactions with modern health systems [30, 31].

Self- reported morbidity measure is symptomatic with healthcare demand and is highly sensitive to social factors that cause health inequalities. Study of inequality in developing nations focuses upon equity in healthcare and healthcare delivery rather than on distribution of health across social and economic subgroups of population and is under-represented. The limited evidence in India so far reveals the pro-rich bias in self-reported morbidity [32–36]. However, most of these studies investigated inequality in the outcome without including horizontal inequity in its purview [37–39] In the absence of such measure, policy prescriptions suggested in these studies should be interpreted with caution. Not only biases prevails in self-reporting morbidity but demand for healthcare captured by untreated morbidity also exhibits high prevalence of socio-economic and systematic inequalities in India that needs further examination.

To encapsulate, the study exposited inequalities and horizontal inequities in self-reported morbidity and untreated morbidity in India*,* the setting and outcomes which are otherwise under-represented in research and policy discourse. These health outcomes in India are characterized by pervasive inequities due to deeply entrenched socio-economic gradients amongst the population. However, the evidence pertaining to these systematic disparities in health is rather scarce in India and does not commensurate with its policy significance. Concomitantly, there is absence of literature on the trajectory of these inequities and the question whether inequities have converged or diverged in India in last few years remains unanswered. Not only the literature in Indian context is limited on horizontal inequities, it is dominated by empirical studies delving into maternal and child health outcomes only. Additionally, there are colossal inter-regional heterogeneities in health outcomes in India, thereby, it is imperative to conduct the analysis at disaggregated and granular level to inform the policy decisions. However, there is a major lacunae in existing studies as regional variations are not captured in most of the studies. Against this backdrop, we attempted to conduct a succinct analysis with the aim to augment the previous studies and overcome gaps in the literature. Our study espouses two-fold objectives:- *Firstly,* we carried out the assessment of magnitude and the change in, horizontal inequities in self-reported health status and untreated morbidity at the national and disaggregated state-level. *Secondly*, we decomposed the income-related inequalities in order to unravel the determinants and contribution of individual factors in driving these inequalities. The focus of this study is on Income-related inequities only as income related inequalities has increased in India during last few decades, Moreover, there is no conclusive evidence on the impact of other socio-economic attribute such as education on health outcomes. In India, not only education related inequalities have been abridged but some evidence suggest that those with less education were more likely to report specific morbidities, sickness and overall poor health [40].

We conducted the analysis on the nationally representative large dataset from three time periods (2004, 2014 and 2017–18), sufficiently apart from one another to analyze the trends using data until most recent round. The decade from 2004 to 2014 was marked by sweeping policy initiatives in Indian healthcare sector and an array of reforms such as National Health Mission and publicly funded health insurance schemes were launched in this period. Post 2014, the government reset the course of Universal Health Coverage through National Health Policy which was further augmented with the launch of Aayushman Bharat initiative in India in 2018. Hence, these study years which are also reflected in our dataset are decisive in unravelling the trajectory and ascertaining the impact of these reforms in our outcome. The latest dataset of 2017–18 is released very recently and is novel in its use in literature. Also, we employed robust Erreyger’s corrected concentration indices to compute the inequities as it is more appropriate measure for bounded variables like the outcome measure in our study. To our knowledge, it is the first study using this modified and appropriate measure for bounded variables in Indian context.

## Data and methods

### Data

Cross sectional unitlevel data was taken from three rounds of nationally representative National Sample Survey Organization surveys: Morbidity and Healthcare (60^th^ round), Survey on Social Consumption (71^st^ round) and Household Social Consumption in India: Health (75^th^ round). These surveys were conducted under the stewardship of Ministry of Statistics and Programme Implementation, Government of India and are representative at the state level as well. It collected information pertaining to households and individuals socio-economic background, morbidity status, utilization of healthcare services and healthcare expenditure on ambulatory, inpatient and delivery care. The survey rounds employed two-stage stratified design, with census villages and urban blocks as the first stage units (FSUs) for rural and urban areas respectively and households as the second stage units (SSUs). The sample size circumscribed 3, 85, 055; 3, 35, 499 and 5, 57, 887 individuals (including death cases) in 60^th^, 71^st^ and 75^th^ rounds respectively.

### Measures

Two outcome measures were gauged in the study: a) Self -reported morbidity and b) Untreated morbidity. The measure of self-reported morbidity was assessed using indicator ‘Whether suffering from any ailment in past 15 days prior to survey?’ and untreated morbidity was defined as individuals not seeking treatment from formal provider upon reporting of an ailment in past 15 days prior to survey using indicator ‘Whether treatment sought on medical advice?’ Both these measures were considered dummy dependent with value 1 defined as ‘reported ailment in past 15 days’ and 0 defined as ‘not reported ailment in past 15 days’ for self- reported morbidity, whereas; for untreated morbidity ‘not sought treatment on medical advice’ was designated the value 1 and ‘sought treatment on medical advice’ was attributed the value 0 for analysis purpose.

An array of household and individual level variables categorized into need/legitimate variables and non-need/illegitimate variables were incorporated in the study to elucidate the determinants associated with inequality of healthcare measure. Need is a rather elusive concept that has been given a variety of interpretations in relation to definition of equity in healthcare delivery [5, 7]. Need or legitimate sources of variation in health are considered to be ethically acceptable and non-need or illegitimate sources are considered to be ethically unfair or unjust, however, the choice of variables are embedded in normative categorization requiring potentially contestable value judgement. Following the literature by the World Bank [41], demographic characteristic of age and gender interaction controlling for gender effect on each age level was used as proxy for need for both outcome variables. Additionally, variable of health condition captured by morbidity status was incorporated for the analysis of untreated morbidity. Non-need variables were selected based on previous studies [15, 41–44], their relevance to understanding inequality and availability within the dataset. The legion of variables comprised of socio-economic characteristics such as education, employment status, social group, religion, quintile groups for wealth status proxied by household monthly per capita expenditure, marital-status, place of residence, insurance coverage, household size and housing conditions score (comprehensive indicator created by coalescing information on latrine access, drinking water source, cooking source, garbage disposal and drainage type using Principal Component Analysis). The complete list of variables and their descriptive statistics are illustrated in Table 1. Monthly per capita consumption expenditure was adjusted accounting for economies of scale in household consumption relating to size and other differences in needs among household members using OECD equivalence scale [45].

### Statistical analysis

Concentration curves and concentration indices were computed to discern inequality in self-reported morbidity and untreated morbidity. Concentration curve plotted the cumulative proportion of health variable (on the vertical axis) against the cumulative proportion of sample (on horizontal axis), ranked by equivalized household monthly per capita expenditure. The standard concentration index, denoted below by C, is computed as twice the area between concentration curve and diagonal and is represented in the formula below [46]:

$$ (1) C=\frac{2}{n\mu}\sum \limits_{i=1}^n{h}_i{R}_i-1 $$

Where, *h*_*i*_ is the variable of interest for the *i*^*th*^ person; *μ* is the mean of *h* and *R*_*i*_ is the *i*^*th*^ ranked individual in socio-economic distribution from most disadvantaged (i.e. poorest) to the least disadvantaged (i.e. richest).

#### Choice of index

The outcome variables are binary characterized by ordinal and bounded nature, therefore, they are not compatible with rank dependent measures such as standard concentration index measuring relative inequality as it doesn’t allow for differences between individuals to be compared. Fundamental predicament facing this variable as with any other binary variable is a) Increase in self-reported and untreated morbidity is mirrored by decrease in unreported and treated morbidity b) An equi-proportionate change in reported/untreated morbidity doesn’t translate into equi-proportionate change in unreported/treated morbidity c) Bounds act as constraints to (proportionally) equal transformations of health variable. Standard concentration index applied to binary variables violates mirror condition as inequality in attainments do not mirror inequality in shortfalls and doesn’t adhere to cardinal invariance property either [47, 48]. Also, a scale invariant, rank dependent inequality index cannot have the property of accounting for relative differences and satisfying mirror condition concomitantly. Thus, in terms of value judgement; an index satisfying mirror property is chosen over index exclusively focusing on relative utilization differences. The mirror condition can only be satisfied by generalized version of modified Concentration Index by Wagstaff and Corrected Erreygers index. Now, the choice between Generalized Index and Erreygers Index depends on value judgements related to desirability of level independence [49]. Since, our variables of interest is at high risk of reporting heterogeneity, Erreyger’s index is the preferred index irrespective of value judgement pertaining to level independence. Relative and absolute inequality can’t be construed in traditional sense for binary variables as bounds of variables act as constraints rendering some of the changes as infeasible. Erreygers by developing notions of ‘quasi-relativity’ and ‘quasi-absoluteness’ mitigates this infeasibility in equi-proportional change or equal additions and is best suited for bounded variables. The Erreygers index is an absolute rather than relative measure and is only rank-dependent inequality measure that has properties of mirror and Quasi-absoluteness [47]. Hence, we chose Erreyger’s Index for our study as it satisfies all desirable properties for rank-dependent indices i.e. *transfer, mirror, level independence* and *cardinal invariance*. The index is formulated as:

$$ \text{E}(\text{h}) = \frac{4{\mu}}{\left( \text{b}_{\text{n}} - \text{a}_{\text{n}} \right)} \text{\text{C}} (\text{h}) $$

Where C (h) represents standard concentration index as denoted in equation 1, μ is the mean of self-reported morbidity and untreated morbidity in the population, a_n_ and b_n_ are the upper and lower bound of outcome variables.

#### Need standardization

The unstandardized distribution of outcome measures doesn’t take into account the fact that demographics and health condition play a role in generating inequality in health. However, these factors can be taken into account by partitioning inequality into avoidable and unavoidable (age-gender) health inequality. Thus, standardization was done to account for differences in population demographic structures to produce refined description of relationship between morbidity status and socio-economic status and to facilitate comparisons across groups. Indirect need standardization method was selected as indirect standardization has greater accuracy when dealing with individual-level data. Non parametric procedure is intuitively selected to estimate variables like self-reported morbidity and untreated morbidity which are binary and non-negative integers. However, evidence suggests that inequity measures doesn’t vary significantly when linear methods are used instead of non-linear methods and an approximation error is found in decomposition with non-linear models [50, 51]. Hence, firstly, we employed linear regression model for standardization represented as:

$$ (3) {y}_i=\alpha +\sum \limits_k{\beta}_k{x}_{ki}+\sum \limits_j{\gamma}_j{z}_{ji}+{\varepsilon}_i $$

Where, *y*_*i*_ is the healthcare outcome for individual *i*; *x*_*ki*_ and *z*_*ji*_ are the vectors of need and non-need determining variables; *α*,*β*_*k*_ and *γ*_*j*_ are the parameters and *ε*_*i*_ is the error term. Secondly, OLS parameter estimates (*α*^^^, *β*^^^_*k*_ and *γ*^^^_*j*_), individual values of need variables (*x*_*ki*_) and sample means of controlled non-need variables (*z*_*j*_^–^) were used to obtain predicted values (x-expected) of self-reported morbidity and untreated morbidity *y*^^^^*x*^_*i*_. Finally, estimates of indirectly standardized outcome variables (*y*^^^^*IS*^_*i*_) were then obtained by subtracting actual and predicted values, plus overall sample mean (*y*^–^) as follows.

$$ (4) {\hat{y}}_i^{IS}={y}_i-{\hat{y}}_i^x+\overline{y} $$

#### Decomposition of index

Decomposition of Erreygers Concentration Index was conducted in order to gauge the relative contribution of covariates in explaining the inequality and residual variation not explained by any factors using methods proposed by Wagstaff et al. [52]. Decomposition was performed using a linear approximation of model based on partial effects of each covariate evaluated at sample means. For any health variable exhibiting a linear relationship with k set of exploratory need variables and z set of exploratory non-need variables as illustrated in eq. 3, the CI for health variable can be decomposed as:

$$ (5) CI=\sum \limits_k\left(\frac{\beta_k{\overline{x}}_k}{\overline{y}}\right){CI}_k+\sum \limits_j\left(\frac{\gamma_j{\overline{z}}_j}{\overline{y}}\right){CI}_j+\frac{GC{I}_{\epsilon }}{\overline{y}} $$

Where, ${\overline{x}}_k$ and ${\overline{z}}_j$ are means of ${x}_k$(need factors) and ${z}_j$ (non-need factors) and *CI*_*k*_ and *CI*_*j*_ represents their respective concentration indices. In the last term (capturing residual); *GCI*_ε_ is the generalized concentration index for *ε*_*i*_ which can be denoted as.

$$ (6) {GC}_{\varepsilon }=\frac{2}{n}\sum \limits_{i=1}^n{\varepsilon}_i{R}_i $$

Which is analogous to the Gini coefficient corresponding to generalized concentration curve and reflects the inequality in health that can’t be explained by systematic variation in other variables.

The formula below is a modified form to decompose Erreyger's index [51].

$$ (7) {E}_c=4\left[\sum \limits_k\left({\beta}_k{\overline{x}}_k\right){CI}_k+\sum \limits_j\left({\gamma}_j{\overline{z}}_j\right){CI}_j+{GCI}_{\varepsilon}\right] $$

Decomposition analysis also facilitated the estimation of horizontal inequity. Horizontal Inequity in the self-reported morbidity and untreated morbidity was calculated by subtracting the absolute contributions made by need/legitimate factors from the unadjusted Erreygers index. A positive (negative value) of HI indicates the inequality in favour of better-off (worse-off) and zero index value indicates that healthcare outcomes and needs are proportionally distributed across income outcomes.

Statistical analysis of data was conducted with STATA 13 statistical software package and weighted^1^ estimates were considered whilst accounting for complex multistage sampling design of surveys. Concentration indices were estimated using the ‘conindex’ command (O’ Donnell, 2016). The confidence interval of horizontal inequity indices were computed using bootstrap method with 1000 replications.

## Results

This section comprises of findings from the analysis which is further disaggregated into various subsections comprising of a) Inequality and Horizontal Inequity in Self-Reported Health Status in India b) Inequality and Horizontal Inequity in Untreated Morbidity in India c) Interregional comparison of Inequality and Horizontal Inequity in Self-Reported Health Status and Untreated Morbidity d) Decomposition of Inequality unravelling determinants and their relative contribution in driving Inequality in India.

### Inequality and horizontal inequity in self-reported health status

**Fig. 1:**
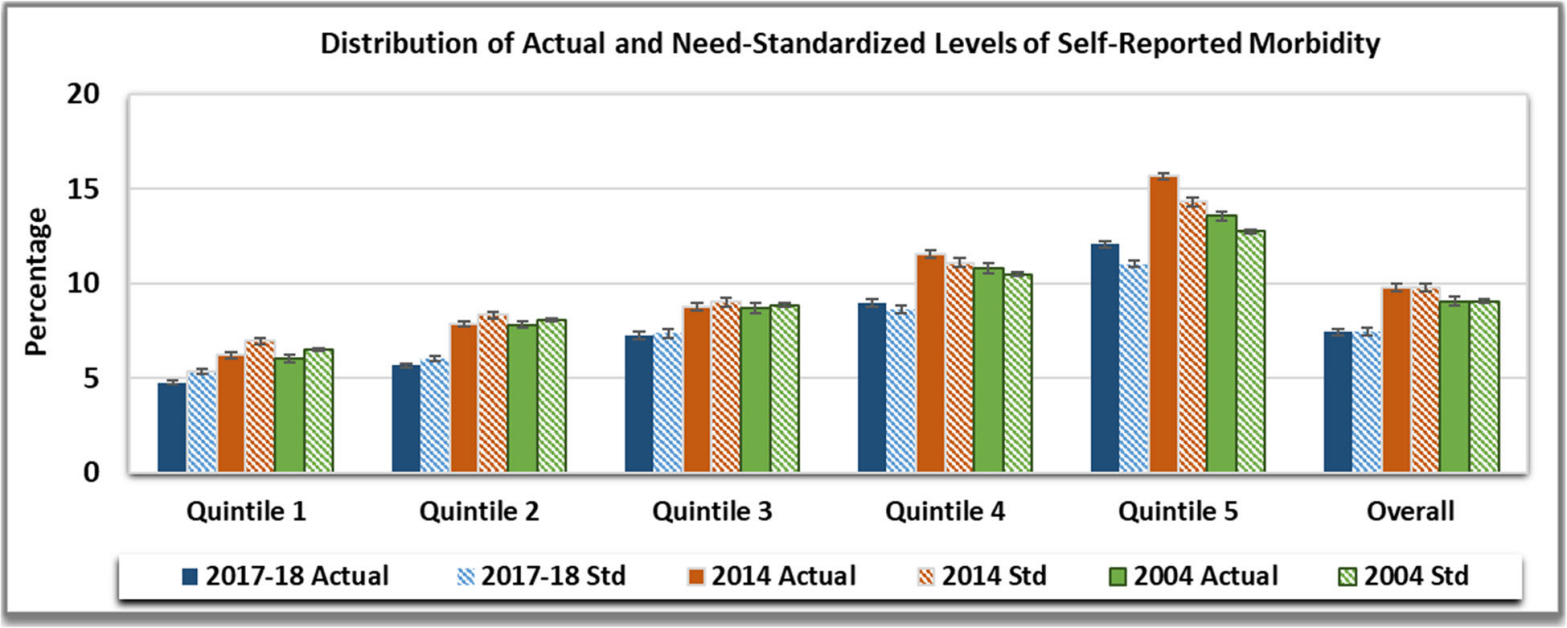
Distribution of Actual and Need-Standardized Levels of Self-Reported Morbidity in India over the study period

Figure 1 encapsulates the actual (unstandardized) and need-standardized distribution of Self-Reported Morbidity over the years. The rate of self-reported morbidity (per ‘000 population) declined from 90.8 in 2004 and 97.9 in 2014 to 74.7 in 2017–18. The mean reporting of morbidity increased in tandem with the increase in relative ranking of quintile groups for all the study years. The largest gradient between lowest and highest quintile group was found in 2014, where, 6.22% (CI 95%: 6.04–6.40) individuals reported being ill, whereas, more than twice as many reported morbidity in the highest quintile group at 15.68% (CI 95%: 14.32–14.06). In 2017–18, for poorest fifth of Indians, the probability of reporting morbidity was 2.1% higher than would be expected on an average, given their need, whereas, richest 20% reported probability of reporting morbidity that was four times greater (8.35%) than the poorest. Whereas, in 2004 and 2014, the need expected distribution was 3% higher for poorest fifth but 3% lower for richest fifth. Estimation of need standardized reported morbidity revealed that standardized reported morbidity was unequally distributed by income. For age-sex standardized income related inequality, the richest quintile (12.75%, CI 95%: 12.51–12.98 in 2004; 14.32, CI 95%: 14.06–14.59 in 2014 and 11.04%, CI 95%: 10.86–11.23 in 2017–18) was estimated to have twice the probability of reporting morbidity than poorest quintile (6.5%,CI 95%: 6.34–6.68 in 2004; 7%, CI 95%: 6.82–7.19 in 2014 and 5.36%, CI 95%: 5.23–5.48 in 2017–18).

The visual inspection of concentration curve with 45 degree line is given in Additional file 1. The concentration curve indicated that for equal need, self-reported morbidity was higher for richer quintiles at all three time points. Curve for self-reported morbidity, which was not being standardized for the confounding effects of age and sex, also revealed that rich reported themselves sick more frequently than the poor. However, dominance cannot be established by simply comparing concentration curve point estimates with diagonal as results are estimated from survey data which is subject to sampling variability; hence, it is imperative to test difference between estimated concentration curve ordinates and diagonal via dominance testing. Both Intersection Union Principle and Multiple Comparison Approach was employed to test the dominance. Both the approaches rejected null of no income related inequality and exhibited that concentration curve is dominated by (lies below) 45 degree line indicating inequality in favor of rich. The corresponding Erreyger’s Concentration Indices and Horizontal Inequity Indices are presented in Table 2. Both Erreyger’s Concentration Indices and Horizontal Inequity Indices for self-reported morbidity was positive and significant for all the study years; connoting that better-off report more morbidity in India. The inequality estimates for 2004 and 2017-18 were at the same level (EI: 0.058; *p*< 0.10 for 2004 and *p*< 0.05 for 2017-18). Whereas, horizontal inequity declined marginally from 2004 (HI: 0.049; *p*< 0.01) to 2017-18 (HI: 0.045; *p*< 0.01).

**Table 1:** Descriptive Statistics

		Self-Reported Morbidity			Untreated Morbidity		
**NEED VARIABLES**							
**Age-Sex Interaction**	**15–29 Male**^**b**^	14.6 (0.35)	14.0 (0.34)	13.3 (0.34)	5.3(0.22)	5.2(0.22)	5.9(0.24)
	**0–14 Male**	14.0 (0.34)	15.4 (0.36)	18.2 (0.38)	10.9 (0.31)	11.9 (0.32)	15(0.36)
	**30–44 Male**	11.1 (0.31)	10.8 (0.31)	10.0 (0.29)	5.7(0.23)	7(0.26)	6.8(0.25)
	**45–59 Male**	8.5(0.27)	7.4(0.26)	6.1(0.24)	10.7 (0.31)	9.8(0.30)	7.6(0.26)
	**60+ Male**	3.4(0.18)	3.8(0.19)	3.5(0.18)	13.8 (0.34)	11.7 (0.32)	12.9 (0.33)
	**0–14 Female**	12.4 (0.32)	13.6 (0.34)	16.6 (0.37)	10.8 (0.30)	9(0.29)	12(0.33)
	**15–29 Female**	13.4 (0.33)	13.0 (0.33)	12.9 (0.33)	5.3(0.22)	7.7(0.27)	5.9(0.24)
	**30–44 Female**	11.0 (0.31)	10.8 (0.31)	9.8(0.29)	5.7(0.23)	11.6 (0.32)	6.8(0.25)
	**45–59 Female**	8.1(0.27)	7.1(0.25)	5.9(0.23)	10.7 (0.31)	13.6 (0.34)	7.6(0.26)
	**60+ Female**	3.5(0.18)	4.0(0.19)	3.5(0.18)	14.2 (0.35)	12.4 (0.33)	13.1 (0.34)
**Duration of Illness**	**Less than equal to 10 days**^**b**^				43.9 (0.50)	44.7 (0.50)	52(0.50)
	**More than 10 days**				56.1 (0.50)	55.3 (0.50)	48(0.50)
**NON-NEED VARIABLES**							
**Marital Status**	**Never Married**^**b**^	44.4 (0.49)	46.0 (0.49)	49.3 (0.49)	27.7 (0.45)	29.2 (0.45)	34.6 (0.48)
	**Currently Married**	50.5 (0.49)	48.6 (0.49)	45.3 (0.49)	56.3 (0.50)	57(0.50)	50.4 (0.50)
	**Widowed/Divorced/Separated**	5.2(0.22)	5.4(0.22)	5.4(0.22)	16(0.37)	13.9 (0.35)	15(0.36)
**Location**	**Rural**	70.5 (0.45)	70.0 (0.45)	74.6 (0.43)	63.6 (0.48)	63.9 (0.48)	71.6 (0.45)
	**Urban**	29.5 (0.45)	30.0 (0.45)	25.4 (0.43)	36.4 (0.48)	36.1 (0.48)	28.4 (0.45)
**Social Group**	**Others**^**b**^	26.4 (0.44)	27.6 (0.44)	31.4 (0.46)	33.9 (0.47)	31.4 (0.46)	37.1 (0.48)
	**Scheduled Tribe**	9.1(0.28)	9.3(0.28)	8.2(0.27)	5.9(0.24)	6.5(0.25)	5(0.22)
	**Other Backward Caste**	44.9 (0.49)	44.3 (0.49)	40.3 (0.49)	42.5 (0.49)	44.4 (0.50)	38.8 (0.49)
	**Scheduled Caste**	19.6 (0.39)	18.8 (0.39)	20.1 (0.40)	17.7 (0.38)	17.7 (0.38)	19(0.39)
**Health Insurance**	**Coverage incl. Govt schemes**^**b**^	15.5 (0.36)	15.2 (0.35)	1.1(0.10)	24.1 (0.43)	23.2 (0.42)	1.8(0.13)
	**No coverage**	84.5 (0.84)	84.7 (0.35)	98.9 (0.10)	75.9 (0.43)	76.8 (0.42)	98.2 (0.13)
**Education**	**Formal schooling Secondary & above**^**b**^	30.3 (0.45)	24.4 (0.42)	13.9 (0.34)	24.5 (0.43)	20.3 (0.40)	12.3 (0.33)
	**Formal schooling till middle**	13.7 (0.34)	13.7 (0.34)	13.5 (0.34)	11.7 (0.32)	12.2 (0.33)	11.3 (0.32)
	**Formal schooling till primary**	28.9 (0.45)	29.3 (0.45)	29.2 (0.45)	27.4 (0.45)	26.7 (0.44)	24.6 (0.43)
	**Literate without formal schooling**	1.0(0.10)	1.0(0.09)	1.0(0.10)	1.2(0.11)	1.3(0.11)	1.3(0.11)
	**Illiterate**	26.1 (0.43)	31.5 (0.46)	42.4 (0.49)	35.1 (0.48)	39.5 (0.49)	50.4 (0.50)
**Employment**	**Wage worker**^**b**^	17.7 (0.38)	18.1 (0.38)	9.7(0.29)	19.9 (0.40)	20.5 (0.40)	10.8 (0.31)
	**Self-employed**	51.1 (0.49)	51.9 (0.49)	51.5 (0.49)	45.9 (0.50)	46.8 (0.50)	48.6 (0.50)
	**Casual laborer**	26.8 (0.44)	25.3 (0.43)	30.5 (0.46)	24(0.43)	23.6 (0.42)	28.5 (0.45)
	**Others**	4.4(0.20)	4.6(0.20)	8.3(0.27)	10.2 (0.30)	9(0.29)	12(0.33)
**Religion**	**Hinduism**^**b**^	81.1 (0.39)	81.1 (0.39)	82.7 (0.37)	77.8 (0.42)	79.3 (0.41)	79.4 (0.40)
	**Islam**	14.1 (0.34)	13.9 (0.34)	12.3 (0.32)	15.1 (0.36)	13.4 (0.34)	13.4 (0.34)
	**Christianity**	2.3(0.14)	2.23 (0.14)	2.1(0.14)	3.8(0.19)	3.8(0.19)	3.8(0.19)
	**Others**	2.5(0.15)	2.7(0.16)	2.9(0.16)	3.3(0.18)	3.5(0.18)	3.4(0.18)
**State Type**	**More Developed(Non-EAG) States**^**b**^	54.1 (0.49)	53.7 (0.49)	55.5 (0.49)	67(0.47)	68.2 (0.47)	64.2 (0.48)
	**Least Developed(EAG)States**	45.9 (0.49)	46.3 (0.49)	45.5 (0.49)	33(0.47)	31.8 (0.47)	35.8 (0.48)
**Housing Conditions Index**	**Low-risk households**^**b**^	41.9 (0.49)	23.0 (0.42)	12.6 (0.33)	43.9 (0.50)	22.5 (0.42)	26.5 (0.44)
	**Medium-risk households**	13.1 (0.33)	18.4 (0.38)	13.7 (0.34)	14.1 (0.35)	24.4 (0.43)	3.8(0.19)
	**High-risk households**	44.9 (0.49)	58.6 (0.49)	73.6 (0.44)	42(0.49)	53.1 (0.50)	69.6 (0.46)
**Household Size**	**Less than 10 members**^**b**^	96.7 (0.17)	96.4 (0.18)	92.8 (0.25)	98(0.14)	97.4 (0.16)	94.6 (0.23)
	**More than 10 members**	3.3(0.03)	3.6(0.18)	7.2(0.25)	2(0.14)	2.6(0.16)	5.4(0.23)
**Expenditure Quintiles**	**Highest MPCE Quintile**^**b**^	17.5 (0.38)	18.2 (0.38)	17.0 (0.37)	18.6 (0.39)	19.1 (0.39)	17.4 (0.38)
	**Fourth MPCE Quintile**	17.8 (0.38)	19.2 (0.39)	17.4 (0.37)	17 (0.38)	18.7 (0.39)	18.1 (0.39)
	**Middle MPCE Quintile**	18.8 (0.39)	19.5 (0.39)	19.6 (0.39)	19.5 (0.40)	20 (0.40)	19.4 (0.40)
	**Second MPCE Quintile**	22.1 (0.41)	20.2 (0.40)	21.9 (0.41)	20.5 (0.40)	19.9 (0.40)	21.1 (0.41)
	**First MPCE Quintile**	23.7 (0.42)	22.9 (0.41)	24.0 (0.42)	24.3 (0.43)	22.3 (0.42)	24(0.43)

Standard error (S.E) in parentheses ;  ^**b**^ denotes the reference category

**Table 2:** Inequality And Horizontal Inequity Indices For India From 2004 To 2017–18

	SELF-REPORTED MORBIDITY			UNTREATED MORBIDITY		
	2004	2014	2017–18	2004	2014	2017–18
**ERREERYG’S CI**	0.058 (.0071)^*^	0.075 (.0028)^**^	0.058 (.0020)^**^	−0.090 (.0008)^***^	− 0.096 (.0011)^***^	− 0.087 (.0098)^*^
**HI INDEX**	0.049 (.0070)^***^	0.058 (.0011)^***^	0.045 (.0008)^***^	−0.103 (.0047)^***^	−0.081 (.0066)^***^	− 0.048 (.0066)^***^

Standard error (S.E) in parentheses

Level of significance: *** *p*<0.01, ** *p*<0.05, * *p*<0.1

### Inequality and horizontal inequity in untreated morbidity

**Fig. 2:**
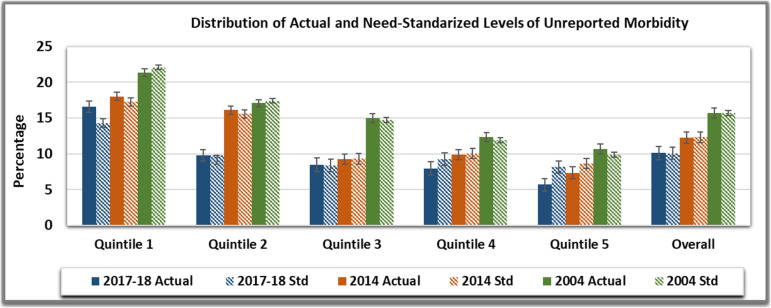
Distribution of Actual and Need-Standardized Levels of Unreported Morbidity in India over the study period

There were 38,803, 33,911 and 43,239 individuals who reported an ailment in last 15 days before the date of survey in 2004, 2014 and 2017–18 respectively. The untreated spells of ailment declined from 15.73% in 2004 to 10.13% in 2017–18. Figure 2 expounds the actual (unstandardized) and need-standardized distribution of untreated morbidity. The untreated morbidity declined with increase in relative ranking of quintile groups. In the highest quintile group, 10.68% (CI 95%: 9.99–11.37) individuals had untreated spell of ailment; whereas 7.37% (CI 95%: 6.75–8) and 5.72% (CI 95%: 5.23–6.22) from this group didn’t seek treatment in 2014 and 2017–18 respectively. The gradient between lowest and highest quintile group in unstandardized distribution did not vary significantly in study period. For poorest of individuals in 2004, the probability of having an untreated spell was 6.37% lower than would be expected on an average given need, whereas, the richest 20% reported the probability of such a condition that is 5.82% higher than the expected. The gap between actual and predicted distribution was lower in the consecutive study periods. Furthermore, need standardized untreated morbidity was also unequally distributed by income. Post standardizing for age-sex and health status, the richest quintile with 9.9%(CI 95%: 9.21–10.59) were one third likely to have untreated spell as compared to the poorest quintile with 22.09%(CI 95%: 21.18–23) untreated spells in 2004. Concomitantly, richest 20% were half as likely as poorest 20% to have untreated morbidity. So, the inequity gradient between these two groups significantly declined over the years from 12.19% in 2004 to 6.07% in 2017–18.

The concentration curve plots (see Additional file 1) exhibited that for equal need, unreported morbidity was higher for poorer quintiles for all study years as concentration curve dominates (lies above) 45 degree line indicating inequality which is disadvantageous to the poor. Further, dominance testing via both Intersection Union Principle and Multiple Comparison Approach rejected the null of no income related inequality. To corroborate, the Erreyger’s Concentration Indices and Horizontal Inequity Indices displayed negative values establishing the pro-poor inequality. The reported difference in inequality between 2004(EI= -0.090, *p*< 0.01) and 2017-18 (EI=-0.87, *p*< 0.10) was marginal. However, significant reduction in horizontal inequity from 2004 (HI= -0.103, *p*< 0.01) to 2017-18 (HI= -0.048, *p*< 0.01) was estimated, indicating the convergence of inequity gap in treatment seeking over the years.

### Inter-state comparison

**Fig. 3:**
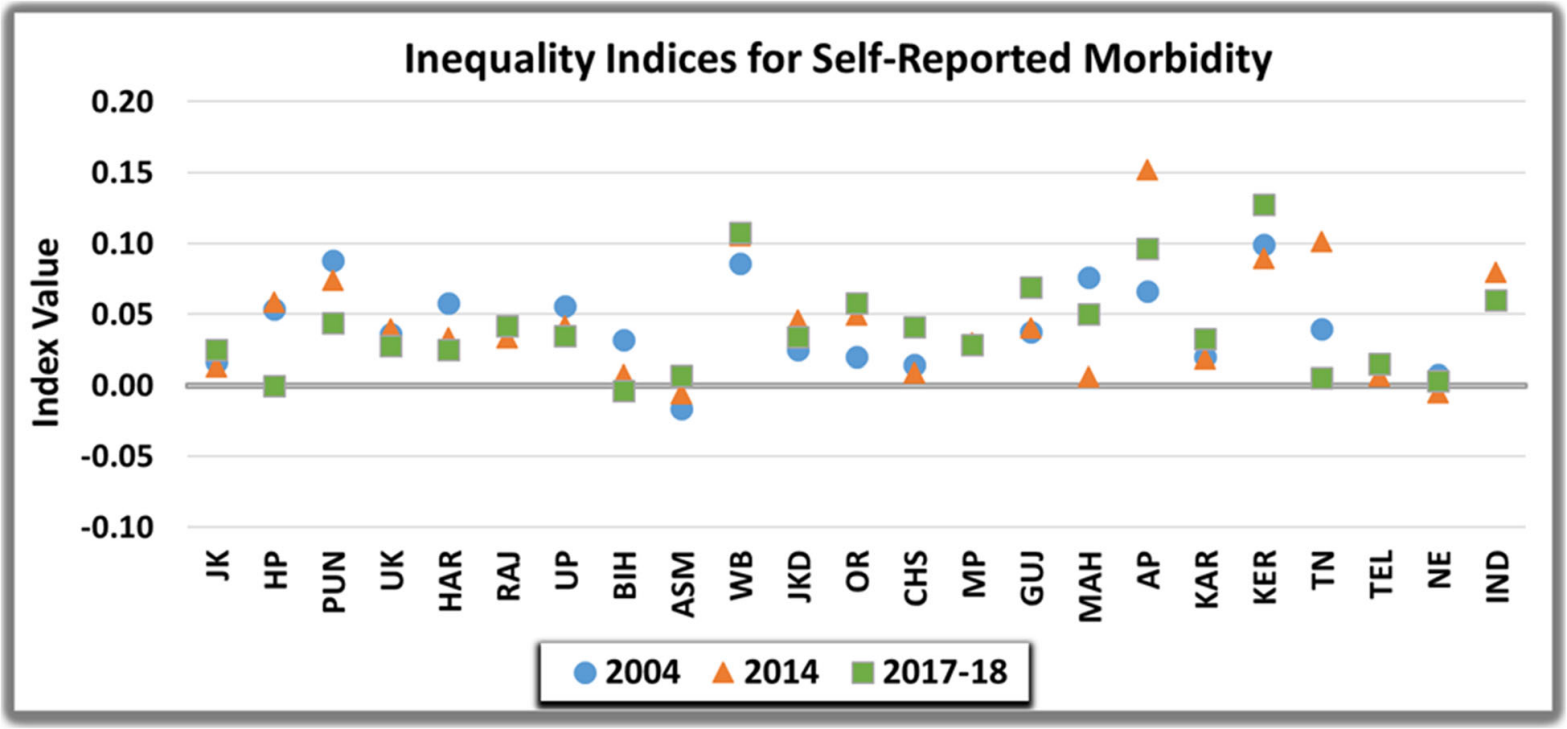
Inequality in the Self-Reported Morbidity in India over the study period

Figure 3 and Fig. 5 represents the extent of income-related inequality pertaining to probability of reporting morbidity and probability of having untreated morbidity across income gradient for major Indian states. The measure of inequality over the years varied across states in India. However, it was perceptibly concentrated amongst the rich for self-reported morbidity and was pro-poor for untreated-morbidity. In more developed states like Andhra Pradesh (EI=0.07, 0.15 and 0.10), Kerala (EI=0.10, 0.09 and 0.13) and West- Bengal (EI=0.09, 0.11 and 0.11) self-reported morbidity in entire study period of 2004, 2014 and 2017-18 was more concentrated amongst richer individuals as compared to all India average. Contrarily, North-Eastern states demonstrated small measures of inequality (EI=0.01 and -0.01) in 2004 and 2014 and perfect equality (EI=0.00) in 2017-18.

**Fig. 4:**
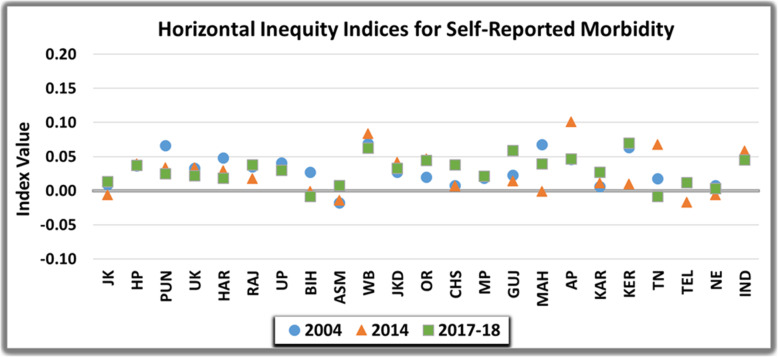
Horizontal Inequity in the Self-Reported Morbidity in India over the study period

Assessment of horizontal inequity post standardizing the differences in need for self-reported morbidity is illustrated in Fig. 4. Horizontal inequity indices for most of the states was positive, indicating that for given need, the better off reported more morbidity. North-eastern states and Jammu and Kashmir evinced lowest estimates of horizontal equity in reporting morbidity during entire study period. None of the EAG states except Chhattisgarh (HI=0.01) exhibited perfect equity or small estimated value of index in 2004. The relative ordering of states changed however, from 2004 to 2017–18 with Bihar attaining an HI value of 0.00 in 2017–18. More developed states of West Bengal (HI-0.09, 0.11 and 0.11), Andhra Pradesh (0.07, 0.15 and 0.10) and Kerala (0.10, 0.09 and 0.13) demonstrated more than all India average estimates of horizontal inequity index over the entire study period in 2004, 2014 and 2017-18 respectively. The value was greater than concentration index implying that need, as proxied by demographic characteristics was more concentrated among higher income groups.

**Fig. 5:**
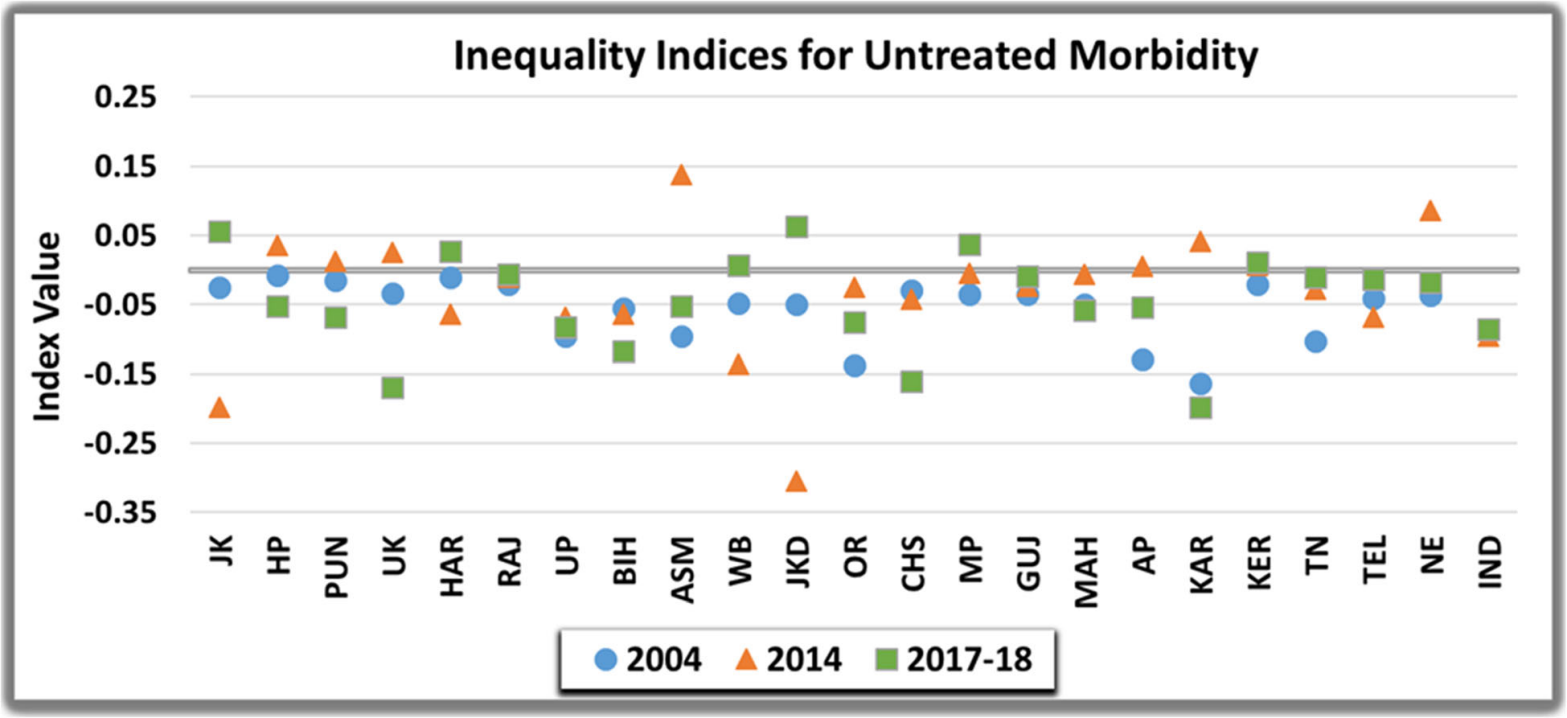
Inequality in the Untreated Morbidity in India over the study period

**Fig. 6:**
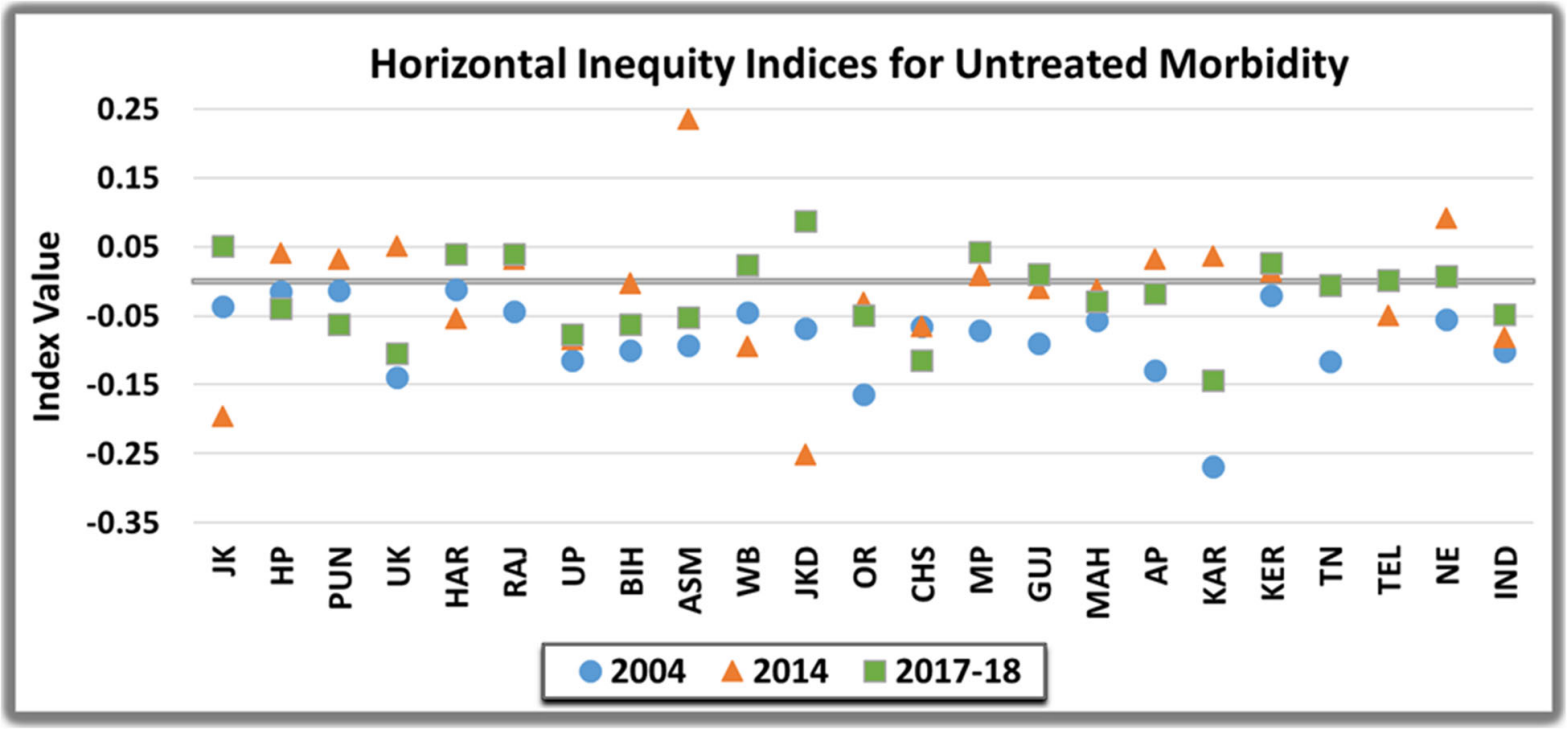
Horizontal Inequity in the Untreated Morbidity in India over the study period

Inter-state comparison of untreated morbidity underscored that inequality was concentrated amongst the poor in majority of Indian states, entailing that poor were more likely to leave the ailment untreated. The inter-state variation is notably elucidated in Fig. 5 and Fig. 6. However, it was transposed from pro-poor to pro-rich in some states such as Jammu and Kashmir, Haryana, West Bengal, Jharkhand, Madhya Pradesh and Kerala from 2004 to 2017-18. The state of Karnataka was most inequitable (EI= − 0.16 and − 0.20) in 2004 and 2017–18 respectively, whereas, Jharkhand (EI= − 0.30) displayed highest inequality in 2014. The measure of horizontal inequity declined from 2004 to 2017–18 in majority of Indian states. Albeit, there was a gradual increase and widening of inequity in Punjab (HI=-0.01 to − 0.06), Himachal Pradesh (HI= − 0.01 to − 0.04) and Chattisgarh (HI= − 0.06 to − 0.12) in this time period. Conversely, the estimates converged towards equal concentration of untreated morbidity amongst higher and lower income groups in North-Eastern states (HI= − 0.05 to 0.01), Gujarat (HI= − 0.09 to 0.01) and Tamil-Nadu (HI= − 0.12 to − 0.01). Subsequently, perfect horizontal equity was achieved in Telangana in 2017-18 (HI= 0.00).

### Decomposition analysis

**Fig. 7:**
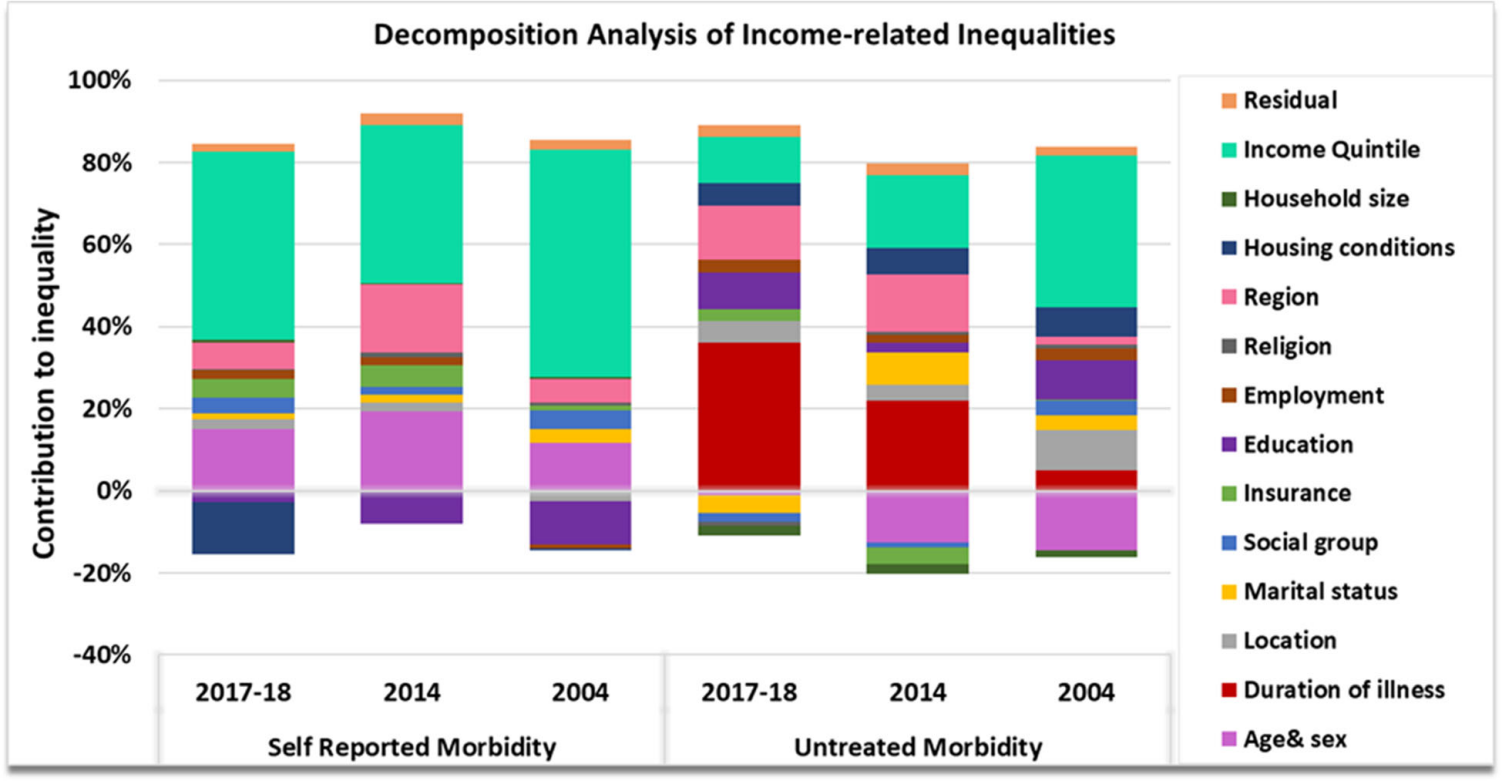
Decomposition Analysis of Income related Inequalities in the Self-Reported Morbidity and Untreated Morbidity in India over the study period

Results of decomposition analysis are summarized in Tables 3 and 4 encapsulating the elasticity (frequency weighted marginal effect computed as marginal effect multiplied by mean of the outcome measure, a constant in the model), concentration index (absolute measure of inequality of contributing factors where negative (positive) value indicates inequality concentrated amongst the worst-off (better-off), absolute contributions (product of elasticity and regressor’s concentration index. A negative (positive) absolute contribution indicates that, if inequality in the outcome variable was determined by that correlate alone, then it would favour the better off (worse-off) and relative contributions (exhibiting how much percentage of inequality in the outcome measure is attributable to the inequality in contributing factor. Relative contribution is computed by dividing its absolute contribution by total inequality of outcome variable and multiplying it by 100) of each determinant and represented in Fig. 7 plotting the aggregate relative contributions of covariates in driving inequality. The descriptive statistics of all the covariates used in the study are elucidated in Table 3. Overall, for self-reported morbidity, the relative contribution of need variables was about 21.4%, 23% and 16.6% of unstandardized indices and the largest contribution to inequality stemmed from illegitimate factors accounting for 78%, 77% and 83% of inequality in 2017-18, 2014 and 2004 respectively. The positive values for legitimate factors indicated that if self-reported morbidity were determined by need alone, it would be pro-rich. Estimated coefficients of linear probability model exhibited that individuals belonging to backward and disadvantaged groups, residing in rural area, not covered under any health insurance scheme, residing in less developed state, having more than 10 members in the household and association with lower income/expenditure quintile groups were less likely to report morbidity. The results demonstrated that over the years majority of pro-rich inequity in self-reported morbidity was caused by illegitimate factors, specifically monthly per capita expenditures. However, the contribution of expenditure declined from 2004 to 2017–18. Rest of the pro-rich inequality embedded in illegitimate factors was circumscribed in a) Level of the development of state b) Insurance cover c) Social Group d) Marital Status and e) Religion for all the study years. Negative contribution was most cogent for housing conditions (− 18% in 2017-18) and education (− 10% and − 15% in 2014 and 2004 respectively) indicating concentration in favor of economically backward sections. The concentration index for aggregate of these categories was also negative reporting pro-poor bias.

**Table 3:** Regression coefficients (b), Absolute contribution and Relative contribution of determinants to income related inequality in Self-reported morbidity in India over the years

		2017–18				2014				2004			
REPORTED MORBIDITY		Coeff	EI	Abs.	% Con	Coeff	EI	Abs.	% Con	Coeff	EI	Abs.	%Con
	***Individuals reporting ailment in last 15 days***												
**Need Variables**													
**Age & Sex Interaction**	15–29 Male^b^												
	0–14 Male	0.036^***^	−0.099	− 0.004	− 0.062	0.041^***^	− 0.109	− 0.004	− 0.059	0.040^***^	− 0.118	− 0.005	− 0.082
	30–44 Male	0.014^***^	0.005	0.000	0.001	0.023^***^	0.011	0.000	0.003	0.014^***^	0.014	0.000	0.003
	45–59 Male	0.059^***^	0.045	0.003	0.046	0.078^***^	0.046	0.004	0.047	0.055^***^	0.041	0.002	0.038
	60+ Male	0.230^***^	0.024	0.006	0.097	0.023^***^	0.031	0.007	0.095	0.234^***^	0.028	0.007	0.112
	0–14 Female	0.030^***^	− 0.099	− 0.003	− 0.052	0.030^***^	− 0.117	− 0.004	− 0.047	0.032^***^	− 0.134	− 0.004	− 0.074
	15–29 Female	0.011^***^	0.007	0.000	0.001	0.022^***^	0.001	0.000	0.000	0.009^***^	0.024	0.000	0.004
	30–44 Female	0.038^***^	0.007	0.000	0.005	0.057^***^	0.013	0.001	0.010	0.035^***^	0.017	0.001	0.010
	45–59 Female	0.092^***^	0.054	0.005	0.086	0.124^***^	0.058	0.007	0.095	0.071^***^	0.045	0.003	0.054
	60+ Female	0.216^***^	0.025	0.005	0.092	0.226^***^	0.028	0.006	0.085	0.220^***^	0.027	0.006	0.101
**SUBTOTAL (NEED)**				**0.012**	**0.214**			**0.017**	**0.230**			**0.009**	**0.165**
**Non-Need Variables**													
**Marital Status**	Never Married^b^												
	Currently Married	−0.002	0.112	0.000	−0.004	0.003	0.130	0.000	0.006	0.012^***^	0.121	0.001	0.024
	Widowed/Divorced	0.040^***^	0.032	0.001	0.022	0.034^***^	0.034	0.001	0.015	0.040^***^	0.033	0.001	0.022
**Location**	Urban^b^												
	Rural	− 0.004^*^	− 0.512	0.002	0.036	− 0.007^*^	− 0.407	0.003	0.039	0.008^**^	−0.425	− 0.003	− 0.057
**Social Group**	Others^b^												
	Scheduled Tribe	−0.025^***^	− 0.102	0.003	0.045	−0.023^***^	−0.098	0.002	0.030	−0.030^***^	−0.095	0.003	0.049
	Other Backward Caste	−0.007^*^	− 0.108	0.001	0.013	0.001	−0.108	0.000	−0.002	− 0.002	− 0.116	0.000	0.003
	Scheduled Caste	− 0.009^***^	− 0.046	0.000	0.007	0.004	−0.046	0.000	−0.003	−0.005	− 0.073	0.000	0.006
**Health Insurance**	Insurance coverage including government schemes^b^												
	No coverage	−0.028^***^	− 0.133	0.004	0.064	−0.040^***^	− 0.121	0.005	0.063	−0.039^***^	− 0.028	0.001	0.019
**Education**	Formal schooling Secondary & above^b^												
	Schooling till middle	0.008	−0.019	0.000	−0.003	0.022^***^	0.005	0.000	0.001	0.018^***^	0.085	0.002	0.026
	schooling till primary	0.006	−0.128	−0.001	− 0.013	0.019^***^	− 0.123	− 0.002	−0.030	0.015^***^	−0.052	− 0.001	− 0.013
	Literate without schooling	0.012	−0.005	0.000	−0.001	0.045^**^	−0.006	0.000	−0.004	0.030^***^	−0.002	0.000	−0.001
	Illiterate	0.008^**^	−0.157	−0.001	− 0.022	0.025^***^	− 0.187	− 0.005	− 0.061	0.033^***^	− 0.295	−0.010	− 0.168
**Employment**	Wage worker^b^												
	Self-employed	−0.002	− 0.102	0.000	0.003	−0.005	− 0.073	0.000	0.004	0.002	−0.026	0.000	−0.001
	Casual laborer	0.000	−0.220	0.000	0.000	0.003	− 0.216	−0.001	− 0.008	0.010^**^	− 0.276	− 0.003	− 0.049
	Others	0.027^***^	0.059	0.002	0.027	0.043^***^	0.049	0.002	0.028	0.020^***^	0.082	0.002	0.028
**Religion**	Hinduism^b^												
	Islam	0.016^***^	−0.047	− 0.001	− 0.013	0.005	− 0.065	0.000	− 0.004	0.016^***^	− 0.050	− 0.001	− 0.013
	Christianity	0.00^***^	0.020	0.000	0.006	0.001^***^	0.020	0.001	0.013	0.001^***^	0.026	0.001	0.022
	Others	0.015^**^	0.034	0.001	0.009	0.014^**^	0.031	0.000	0.006	−0.001	0.028	0.000	0.000
**State Type**	More Developed(Non-EAG) States^b^												
	Least Dev. (EAG) States	−0.011^***^	− 0.512	0.005	0.094	−0.033^***^	− 0.450	0.015	0.198	−0.012^***^	− 0.381	0.005	0.078
**Housing Conditions Index**	Low-risk households^b^												
	Medium-risk households	0.018^***^	0.026	0.000	0.008	0.006	0.168	0.001	−0.046	0.005	0.239	0.001	0.020
	High-risk households	0.022^***^	−0.487	− 0.011	− 0.189	0.006	− 0.539	− 0.003	0.014	−0.005	− 0.516	0.003	0.046
**Household Size**	Less than 10 members^b^												
	More than 10 members	−.0.009^**^	− 0.067	0.001	0.011	−0.001	− 0.069	0.000	0.001	−0.003	− 0.124	0.000	0.007
**Expenditure Quintiles**	Highest MPCE Quintile^b^												
	Fourth MPCE Quintile	−0.022^***^	0.336	−0.007	− 0.130	− 0.026^***^	0.341	− 0.009	− 0.116	− 0.025^***^	0.338	− 0.008	− 0.143
	Middle MPCE Quintile	− 0.033^***^	0.079	−0.003	− 0.045	− 0.041^***^	0.044	− 0.002	− 0.024	− 0.041^***^	0.090	− 0.004	− 0.063
	Second MPCE Quintile	− 0.045^***^	− 0.270	0.012	0.211	−0.043^***^	− 0.275	0.012	0.158	−0.047^***^	− 0.264	0.012	0.211
	First MPCE Quintile	−0.050^***^	− 0.723	0.036	0.620	−0.049^***^	−0.706	0.035	0.460	−0.060^***^	− 0.729	0.044	0.745
**SUBTOTAL (NON-NEED)**				**0.043**	**0.778**			**0.056**	**0.769**			**0.047**	**0.835**
**Residual**		0.002^***^	0.002	0.002	0.028	0.002^***^	0.002	0.002	0.033	0.002^***^	0.002	0.002	0.035
**EI (Unstandardized)**				**0.058**	**1.000**			**0.075**	**1.000**			**0.058**	**1.000**

Standard error (S.E) in parentheses

Level of significance: *** *p*<0.01, ** *p*<0.05, * *p*<0.1

**Table 4:** Regression coefficients (b), Absolute contribution and Relative contribution of determinants to income related inequality in untreated morbidity in India over the years

		2017–18				2014				2004			
UNTREATED MORBIDITY		Coeff	EI	Abs.	% Con	Coeff	EI	Abs.	% Con	Coeff	EI	Abs.	%Con
	***Individuals reporting ailment and not availing treatment in last 15 days.***												
**Need Variables**													
**Age & Sex Interaction**	15–29 Male^b^												
	0–14 Male	− 0.053^**^	− 0.157	0.008	− 0.096	− 0.073^**^	− 0.134	0.010	− 0.102	− 0.082^***^	− 0.135	0.011	− 0.122
	30–44 Male	0.000	0.012	0.000	0.000	0.068^*^	−0.012	− 0.001	0.008	− 0.018	− 0.017	0.000	− 0.004
	45–59 Male	−0.020	0.048	−0.001	0.011	0.024	0.059	0.001	−0.015	−0.008	0.042	0.000	0.004
	60+ Male	−0.034	0.049	−0.003	0.037	0.019	0.081	0.001	−0.015	0.038^**^	0.073	0.003	−0.031
	0–14 Female	−0.037	− 0.123	0.005	− 0.053	−0.051^*^	− 0.097	0.005	− 0.051	−0.062^***^	− 0.127	0.008	− 0.088
	15–29 Female	−0.022	− 0.043	0.001	− 0.011	−0.003	− 0.031	0.000	− 0.001	0.000	− 0.012	0.000	0.000
	30–44 Female	−0.015	−0.007	0.000	−0.001	0.032	−0.017	− 0.001	0.006	− 0.025	0.007	0.000	0.002
	45–59 Female	−0.048	0.085	−0.004	0.047	0.026	0.100	0.003	−0.027	−0.039^**^	0.075	−0.003	0.033
	60+ Female	−0.042	0.107	−0.005	0.052	0.026	0.053	0.001	−0.014	0.007	0.088	0.001	−0.006
**Duration of Illness**	Less than equal to 10 days^b^												
	More than 10 days	−0.113^***^	0.356	−0.040	0.463	−0.134^***^	0.268	−0.036	0.371	−0.030^***^	0.215	−0.006	0.071
**SUBTOTAL (NEED)**				**−0.039**	**0.449**			**−0.015**	**0.159**			**0.013**	**−0.142**
**Non-Need Variables**													
**Marital Status**	Never Married^b^												
	Currently Married	0.011	0.211	0.002	−0.028	−0.057^**^	0.161	−0.009	0.095	−0.038^**^	0.167	−0.006	0.071
	Widowed/Divorced	0.026	0.089	0.002	−0.027	− 0.062^**^	0.056	− 0.003	0.036	0.025	0.062	0.002	−0.017
**Location**	Urban^b^												
	Rural	0.012	−0.498	− 0.006	0.066	0.014	−0.438	− 0.006	0.064	0.031^***^	−0.422	− 0.013	0.146
**Social Group**	Others^b^												
	Scheduled Tribe	−0.007	−0.088	0.001	−0.008	− 0.008	−0.094	0.001	−0.008	0.035^**^	−0.070	− 0.002	0.027
	Other Backward Caste	−0.010	−0.134	0.001	−0.016	− 0.006	−0.130	0.001	−0.007	0.020^**^	−0.137	− 0.003	0.030
	Scheduled Caste	−0.004	−0.082	0.000	−0.004	− 0.020^**^	−0.024	0.000	−0.005	− 0.003	−0.093	0.000	−0.003
**Health Insurance**	Insurance coverage including government schemes^b^												
	No coverage	0.015^**^	−0.218	−0.003	0.038	−0.039^***^	− 0.169	0.007	− 0.069	0.008	− 0.046	0.000	0.004
**Education**	Formal schooling Secondary & above^b^												
	Schooling till middle	0.010	0.021	0.000	−0.002	0.031^**^	0.048	0.001	−0.015	−0.002	0.080	0.000	0.001
	Schooling till primary	0.034^**^	−0.080	−0.003	0.031	0.028^**^	−0.043	−0.001	0.013	0.019^*^	0.015	0.000	−0.003
	Literate without schooling	0.041	−0.004	0.000	0.002	0.033	−0.012	0.000	0.004	0.023	0.002	0.000	−0.001
	Illiterate	0.028^**^	−0.252	−0.007	0.081	0.014	−0.285	−0.004	0.041	0.036^***^	−0.354	−0.013	0.143
**Employment**	Wage worker^b^												
	Self-employed	0.005	−0.175	− 0.001	0.011	0.021^**^	−0.122	− 0.003	0.027	− 0.015	−0.052	0.001	−0.008
	Casual laborer	0.009	−0.230	−0.002	0.023	0.023^*^	−0.218	−0.005	0.053	0.008	−0.288	−0.002	0.027
	Others	−0.004	0.209	−0.001	0.009	0.038^**^	0.125	0.005	−0.049	−0.016	0.127	−0.002	0.023
**Religion**	Hinduism^b^												
	Islam	−0.020^**^	−0.075	0.002	−0.017	0.018	−0.068	− 0.001	0.013	0.003	−0.080	0.000	0.003
	Christianity	0.001	0.058	0.001	−0.009	0.000	0.046	0.000	−0.003	0.002^**^	0.051	0.002	−0.017
	Others	−0.038^**^	0.031	−0.001	0.013	−0.009	0.020	0.000	0.002	−0.065^***^	0.043	−0.003	0.031
**State Type**	More Developed(Non-EAG) States^b^												
	Least Dev. (EAG)States	0.031^***^	−0.466	−0.014	0.167	0.059^***^	−0.386	−0.023	0.236	0.007	−0.362	−0.003	0.028
**Housing Conditions**	Low-risk households^b^												
	Medium-risk households	0.012	0.012	0.000	−0.002	−0.007	0.146	−0.001	0.011	−0.004	0.019	0.000	0.001
	High-risk households	0.016^*^	−0.411	− 0.006	0.075	0.017	−0.545	− 0.009	0.095	0.019^**^	−0.512	− 0.010	0.106
**Household Size**	Less than 10 members^b^												
	More than 10 members	−0.051^**^	−0.054	0.003	−0.032	− 0.061^**^	−0.062	0.004	−0.039	− 0.025	−0.094	0.002	−0.026
**Expenditure Quintiles**	Highest MPCE Quintile^b^												
	Fourth MPCE Quintile	0.000	0.311	0.000	0.000	0.004	0.322	0.001	−0.013	−0.006	0.341	−0.002	0.023
	Middle MPCE Quintile	−0.015	0.072	−0.001	0.012	−0.016	0.035	−0.001	0.006	0.007	0.075	0.001	−0.006
	Second MPCE Quintile	−0.019*	−0.253	0.005	−0.057	0.032	−0.283	− 0.009	0.094	0.023^**^	−0.262	− 0.006	0.067
	First MPCE Quintile	0.022^*^	−0.736	−0.016	0.188	0.030^**^	−0.692	−0.020	0.212	0.057^***^	−0.729	−0.042	0.462
**SUBTOTAL (NON-NEED)**				**−0.044**	**0.515**			**−0.076**	**0.792**			**−0.100**	**1.111**
**Residual**		−0.003	−0.003	− 0.003	0.036	− 0.005^*^	−0.005	− 0.005	0.049	− 0.003^***^	−0.003	− 0.003	0.030
**EI (Unstandardized)**				**−0.087**	**1.000**			**−0.096**		**1.000**		**−0.090**	**1.000**

Standard error (S.E) in parentheses

Level of significance: *** *p*<0.01, ** *p*<0.05, * *p*<0.1

Untreated morbidity exhibited increase in contribution of legitimate variables in explaining inequality. The relative contribution was − 14.2% in 2004 with positive index value (0.013) weighing down the overall pro-poor inequality which was increased to 44.9% with the absolute aggregate contribution of − 0.04 (pro-poor concentration) in 2017–18. However, the relative contribution of legitimate variables was disparate; while, duration of illness contributed to pro-poor inequality; age-sex interaction sought to reduce inequality for all the years. The pro-rich pull exerted by age-sex was more than counteracted by pro-poor effect of illness in 2017–18 and 2014. Amongst the illegitimate factors, most of the inequality was attributed to expenditure quintiles in 2004 and 2014 (54.6 and 29.9% respectively). The contribution of expenditure declined in generating the inequality in 2017-18, where, level of development of state was the biggest contributor amongst the factors amenable to policy intervention. Factors such as belonging to rural area, disadvantaged social group and poorest expenditure quintile, having poor housing conditions and being illiterate were positively associated with untreated morbidity in 2004. Whereas, in 2014 and 2017–18; lack of insurance coverage, less education level and residing in less developed state exhibited positive and significant (*p*< 0.05) relationship with untreated morbidity. Whereas, being a male in 0-14 age group and suffering for more than 10 days from the illness significantly reduced the likelihood of having untreated morbidity in all the years.

## Discussion and conclusion

Our study found systematic inequalities in self-reported morbidity and untreated morbidity. Self- reported morbidity rates were more amongst the richer with significant inequality gap between poorest and richest quintile. Both Erreyger’s concentration index and Horizontal Inequity index was positive denoting inequalities in favour of the rich. These findings were analogous to studies conducted in other countries such as Thailand, [24], South Africa [53] and Chile [42] where inequality gradients were found to be disadvantageous to the poor. It can be construed that self-reported morbidity is subjective measure with a certain degree of perception bias which is pervasive in these type of surveys where cognitive processes, social desirability and survey conditions can alter interviewee’s response. It can further be elucidated with Sen’s argument where he asserted that positional objectivity (in terms of income and education etc.) influences perception of disease and decision to self-report. He further annotated that socially disadvantaged individuals fail to perceive and report the presence of illness or health-deficits because an individual’s assessment of their health is directly contingent on their social experience [54]. Further, McMullen and Luborsky explained self-rated health appraisals as cultural and identity process in an ethnographic study of African American Elders [55]. They succinctly explained that self-rated health integrates a cultural process of identity formation, whereby, identities are multiple, simultaneously, individual and collective, and produced within particular socio-economic and historical formation. Detection bias also significantly affect rate of diagnosis between different wealth groups as wealth is often correlated with education status and access to healthcare. Our results also highlighted the role of having residence in rural area and absence of insurance cover in driving inequities which can also arise due to organizational barriers in detecting the morbidities. As the measure of morbidity is self-reported, it is sensitive to internal frame of reference and response styles. Hence, reporting heterogeneity can arise due to Positional Effect (Response shift) or Dispositional Effect/Judgement Effect. Heterogeneity in reporting due to attenuation bias arising from measurement error can be minimized with the help of Anchoring Vignettes using hypothetical stories or description of health problems which can then be adjusted and corroborated with individual’s subjective assessment of own situation [56]. Additionally, a multiple question instrument based on disease symptoms is recommended to reduce biases.

There was a marked heterogeneity in the magnitude of inequality and horizontal inequity amongst the states. Jammu and Kashmir and North Eastern states divulged lowest inequity in self-reported morbidity rates for entire study period, whereas, more developed states like Kerala, Andhra Pradesh and West Bengal had more pronounced inequity in 2017–18. These states are also identified to be at advanced stages of epidemiological transition level (which is defined on the basis of ratio of Disability-Adjusted Life Years (DALYs), computed as the sum of years of potential life lost due to premature mortality and the years of productive life lost due to disability)from communicable disease to those from non-communicable disease and injuries combined) with burden of disease disproportionately skewed towards non-communicable diseases such as heart disease, diabetes, respiratory problems and cancer [57, 58]. Literature has established that reporting of morbidity is lower for non-communicable and chronic diseases vis. a vis. other disease conditions amongst poorer sections as ignoring minor symptoms/early signs of chronic diseases and detection bias is stronger for Non-Communicable Diseases (NCD’s) [21]. Indian evidence evinced that self-reported diagnosed cases of disease prevalence for NCDs were significantly higher in most affluent quintile compared with least affluent quintile [59]. In a study conducted in Kerala region of India [60], it was found that inequality ratio between poorest and richest quintile for reporting chronic diseases was twice for all ailments and 2.4 times for acute ailments highlighting greater inequality in reporting of NCD’s and chronic conditions. Conversely, disease prevalence measured using standardized measures in India for chronic conditions tended to show either negative/no association with wealth, indicating probable under-diagnosis and under-reporting of diseases among lower socio-economic status groups [58]. To encapsulate the argument, it is understood that for most of the NCD conditions, diagnosis at health facility is the basis for the knowledge of presence of condition. This self-reporting, aided by facility-based diagnosis may transmute to an underestimation amongst poor because of their relatively low uptake and utilization of health services and greater likelihood of suffering from undiagnosed illness. Reporting for infectious disease is however, more as it is easily observable and experienced even without formal diagnosis; therefore, influencing equity considerations depending on epidemiological profiles of states.

Untreated morbidity in all three study years was estimated to have pro-poor inequality and inequity as mirrored in negative values of Erreyger’s concentration index and Horizontal Inequity index. Although, there were favourable changes in un-treated morbidity between three time periods, there still remained considerable inequities that were disadvantageous to the poor. The results are congruous with other studies in Indian setting where distribution of sampled untreated ailing person was pro-poor [16, 61]. The reasons cited by the individuals for not seeking treatment were multifold that can be understood using Penchansky’s framework of access and utilization which is embedded in five dimensions i.e. Availability, Accessibility, Affordability, Acceptability and Accommodation [62]. In 2004, affordability (29.62%) and spatial accessibility which is interaction of accessibility and availability (10.56%) constituted important barriers to seek treatment. Albeit, health seeking behavior was mostly influenced by the reason of not considering the ailment serious (39.56%). In subsequent years, there was significant decline in impediment related to costs and affordability which was 5.99% in 2014 and 5.24% in 2017–18. However, there has been a mercurial rise in the proportion of individuals stating that ailment was not serious to merit care in 2014 (61.94%) and 2017–18(73.51%). The relative contribution of supply side barriers such as spatial accessibility declined by half in 2017–18. Previous literature in Indian context suggests that financial barriers remained Achilles heel to seek treatment for poor, whereas, perception of severity of ailment was prominent reason for richer individuals [17, 61]. However, demand for healthcare services in recent years which has been driven by perceived disease severity is also susceptible to inequality gradients stemming from gender, social status and education [63, 64]. It has a serious policy implication as although, health-seeking behavior is concurrently influenced by demand and supply side constraints requiring impetus to both demand and supply; in resource constraint setting, selection of appropriate interventions such as behavioral change communication to alter the attitudes embedded in self-medication at the onset of illness and seeking care from qualified medical professional only when conditions deteriorate is required. Concomitantly, strengthening primary healthcare network targeting the socially and economically disadvantaged needs to be prioritized.

Further, untreated morbidity declined gradually over the years which can be attributed to interventions and reforms by Indian government to provide affordable and quality care. In the past decade, India introduced many publicly funded health insurance schemes (PFHIs) like Rashtriya Swasthya Bima Yojana (RSBY), myriad state health insurance schemes and more recently Ayushman Bharat Yojana improving the treatment seeking. Strengthening public health systems through National Health Mission has given impetus to care seeking at public facilities in India. Utilization for ambulatory care in public facilities increased in 2014 from 2004 by 6% and further to 4.6% in 2017–18 from 2014. Similar trend was exhibited for hospitalizations where, public facilities use increased by 13% from 2004 to 2017–18. Moreover, latest estimates affirms that poor are going more to the public facilities as 35.9% from poorest quintile visited public facilities for outpatient care in 2017–18 as compared to the 19.7% from richest quintile. We therefore, posit that reforms in public health sector in India contributed in reducing the rates and inequities in untreated morbidity over the years. However, there is need of richer datasets than the ones existing if we are to constitute reliable evidence to inform policy-making.

There are few caveats emanating from nature of data in this study and results should be interpreted with caution. Firstly, this dataset doesn’t circumscribe any objective measure of health/vignette schedule making it difficult to gauge the relative contribution of actual increase in disease burden and subjective perception bias in the levels of reported illness. Secondly, only individual and household level determinants were incorporated to explain self-reported morbidity and untreated morbidity, whereas, other factors pertaining to health system reforms, culture and behavior is not in the preview of this study due to data constraints. Thirdly, the information on outcomes and determinants was collected concurrently due to cross-sectional design, thus, associations rather than causal relationships are defined in the study.

## Supplementary Information

**Additional file 1.**

## Data Availability

The datasets generated/or analyzed during the current study are available from Ministry of Statistics and Programme Implementation (MOSPI), Government of India upon request.

## Footnotes

1 NSS = Number of first stage units surveyed within a substratum for the sub-sample NSC = Number of first stage units surveyed within a substratum for subsample combined MLT= Weight or multiplier within a substratum for the sub-sample Final weight= MLT/100, if NSS=NSC = MLT/200 otherwise # Analytical and Probability weights used in the analysis # For details on the weighting strategy and formulae for multipliers, refer Instruction manual, NSSO Round 60, 71 and 75 retrieved from: http://mospi.nic.in/schedule-instructions
