# Correction to: Horizontal inequity in self-reported morbidity and untreated morbidity in India: Evidence from National Sample Survey Data

**DOI:** 10.1186/s12939-021-01469-4

**Published:** 2021-05-31

**Authors:** Veenapani Rajeev Verma, Umakant Dash

**Affiliations:** Department of Humanities and Social Sciences, Indian Institute of Technology Madras, Chennai, India

**Correction to: Int J Equity Health 20, 49 (2021)**

**https://doi.org/10.1186/s12939-020-01376-0**

Following publication of the original article [1], we have been notified that parts of text were deranged. The text parts should read as follows:

**INTRODUCTION**

Besides, the index value based on income level and wealth was colossally high at 0.54 and 0.75 in 2011–12. Further, the share of national income accruing to top 1% income earners was 22% in 2014 [9], which remained unvaried in 2018. As per the latest estimates, in 2018, top 1% captured 21.4% and top 10% garnered 56.1% of income in India [10]. Evidence on socio-economic inequalities in access and utilization of healthcare services is also ubiquitous in India, albeit the literature in Indian context is dominated by empirical studies delving into maternal and child health outcomes only [11–14].

Therefore, an analysis of extent, trends and determinants of horizontal inequities in the prevalence of morbidity and utilization of health services at regional level needs to be undertaken.

Furthermore, self-rating of health embodies complex human judgement about severity of current illness and provides a dynamic evaluation discerning both the trajectory and current level of health [22].

The seminal work by Idler and Benyamini,1997 [23] reviewed a set of studies and found out that in great majority of cases; self-ratings adds something more to the prediction of mortality and concluded that self-ratings represent a source of valuable data on health status presenting an indispensable dimension of health status, without which individual health status cannot be assessed. There is a good basis for using self-rated health as an outcome as it can provide more holistic view of health which may not be reflected in objective measures such as those based on specific medical diagnosis [24]. Self-rated health influences behavior that subsequently affect health status. It is pertinent to investigate the inequities in self-reported morbidities as heterogeneities in perceptions subsequently, influences the health seeking behavior. It is desirable to examine a dynamic rather than static perspective on health as is subsumed in self-reported measures. Furthermore, objective measures such as consultation data has some constraints e.g. it does not reflect all the health problems in a population since many of those are not bought to attention of healthcare services [25]. Subjective measures such as self-reported health status on the other hand, is extremely valuable measure of health as it gauges what really matters and is an indicator of patient’s empowerment [26]. While assessing a person’s health condition and health-care demand, it is essential to take perception of individual about his/her health into consideration; hence, self-reported health status represents a summary statement about how numerous aspects of health, both subjective and objective are combined within perceptual framework of individual respondent [27]. Some evidence purported that self-reported health could also predict hospitalization and specialist consultation better than diagnosed health conditions [26]. From the sociological perspective, it is argued that self-reported illness represents well-being of an individual more than an objective, medically-confirmed disease [28]. Self-reports has been used profusely in developing countries using large-scale demographic and health surveys (DHS) for estimating prevalence of illnesses and remains one of the most widely used methods in clinical, public health, social and economic research [29]. Specifically, in a country like India, self-reported measure is both desirable and feasible as objective data on health is scarce whereas, self-reported measures are easy and inexpensive to collect and studies have also demonstrated them to be a good predictor of mortality and functionability, even after controlling for other objective health measures. Evidence from other low income setting of Bangladesh demonstrates both the multidimensional nature and effective predictive power of relatively simple and low-cost measure of self-reported health and establishes its validity and supports the notion that individuals can effectively assess their own health status even in settings of poor education and lower level of interactions with modern health systems [30, 31].

Concomitantly, there is absence of literature on the trajectory of these inequities and the question whether inequities have converged or diverged in India in last few years remains unanswered.

Moreover, there is no conclusive evidence on the impact of other socio-economic attribute such as education on health outcomes. In India, not only education related inequalities have been abridged but some evidence suggest that those with less education were more likely to report specific morbidities, sickness and overall poor health [40].

**DATA AND METHODS:**

It collected information pertaining to households and individuals socio-economic background, morbidity status, utilization of healthcare services and healthcare expenditure on ambulatory, inpatient and delivery care. The survey rounds employed two-stage stratified design, with census villages and urban blocks as the first stage units (FSUs) for rural and urban areas respectively and households as the second stage units (SSUs). The sample size circumscribed 3, 85, 055; 3, 35, 499 and 5, 57, 887 individuals (including death cases) in 60th, 71st 75th rounds respectively.

The complete list of variables and their descriptive statistics are illustrated in Table 1.

1 $$ C=\frac{2}{n\mu}\sum \limits_{i=1}^n{h}_i{R}_i-1 $$

“Where, *h*_*i*_ is the variable of interest for the *i*^*th*^ person; *μ* is the mean of *h* and *R*_*i*_ is the *i*^*th*^ ranked individual in socio-economic distribution from most disadvantaged (i.e. poorest) to the least disadvantaged (i.e. richest).

**Data AND Methods: Choice of Index**

Standard concentration index applied to binary variables violates mirror condition as inequality in attainments do not mirror inequality in shortfalls and doesn’t adhere to cardinal invariance property either [47, 48]. Also, a scale invariant, rank dependent inequality index cannot have the property of accounting for relative differences and satisfying mirror condition concomitantly. Thus, in terms of value judgement; an index satisfying mirror property is chosen over index exclusively focusing on relative utilization differences. The mirror condition can only be satisfied by generalized version of modified Concentration Index by Wagstaff and Corrected Erreygers index. Now, the choice between Generalized Index and Erreygers Index depends on value judgements related to desirability of level independence [49].

2 $$ \mathrm{E}\ \left(\mathrm{h}\right)=\frac{4\upmu}{\left({\mathrm{b}}_{\mathrm{n}}-{\mathrm{a}}_{\mathrm{n}}\right)}\ \mathrm{C}\ \left(\mathrm{h}\right) $$

“Where C (h) represents standard concentration index as denoted in eq. 1, μ is the mean of self-reported morbidity and untreated morbidity in the population, a_n_ and b_n_ are the upper and lower bound of outcome variables.

**Data AND Methods: Need Standardization**

Hence, firstly, we employed linear regression model for standardization represented as:

3 $$ {y}_i=\alpha +\sum \limits_k{\beta}_k{x}_{ki}+\sum \limits_j{\gamma}_j{z}_{ji}+{\varepsilon}_i $$

“Where, *y*_*i*_ is the healthcare outcome for individual *i*; *x*_*ki*_ and *z*_*ji*_ are the vectors of need and non-need determining variables; *α*, *β*_*k*_ and *γ*_*j*_ are the parameters and *ε*_*i*_ is the error term. Secondly, OLS parameter estimates ($\hat{\alpha}$, ${\hat{\beta}}_k$ and ${\hat{\gamma}}_j$), individual values of need variables(*x*_*ki*_) and sample means of controlled non-need variables(${\overline{z}}_j$) were used to obtain predicted values(x-expected) of self-reported morbidity and untreated morbidity ${\hat{y}}_i^x$. Finally, estimates of indirectly standardized outcome variables $\left({\hat{y}}_i^{IS}\right)$ were then obtained by subtracting actual and predicted values, plus overall sample mean ($\overline{y}$) as follows.

**Data AND Methods: Decomposition of index**

5 $$ CI=\sum \limits_k\left(\frac{\beta_k{\overline{x}}_k}{\overline{y}}\right){CI}_k+\sum \limits_j\left(\frac{\gamma_j{\overline{z}}_j}{\overline{y}}\right){CI}_j+\frac{GCI_{\epsilon }}{\overline{y}} $$

“Where, ${\overline{x}}_k$ and ${\overline{z}}_j$ are means of *x*_*k*_ (need factors) and *z*_*j*_ (non-need factors) and *CI*_*k*_ and *CI*_*j*_ represents their respective concentration indices. In the last term (capturing residual); *GCI*_*ϵ*_ is the generalized concentration index for *ε*_*i*_ which can be denoted as.

**RESULTS**

This section comprises of findings from the analysis which is further disaggregated into various subsections comprising of a) Inequality and Horizontal Inequity in Self-Reported Health Status in India b) Inequality and Horizontal Inequity in Untreated Morbidity in India c) Inter-regional comparison of Inequality and Horizontal Inequity in Self-Reported Health Status and Untreated Morbidity d) Decomposition of Inequality unravelling determinants and their relative contribution in driving Inequality in India.

**RESULTS: Inequality And Horizontal Inequity In Self-Reported Health Status**

The inequality estimates for 2004 and 2017–18 were at the same level (EI: 0.058; *p* < 0.10 for 2004 and *p* < 0.05 for 2017–18). Whereas, horizontal inequity declined marginally from 2004 (HI: 0.049; *p* < 0.01) to 2017–18 (HI: 0.045; *p* < 0.01).

**RESULTS: Inequality And Horizontal Inequity In Untreated Morbidity**

To corroborate, the Erreyger’s Concentration Indices and Horizontal Inequity Indices displayed negative values establishing the pro-poor inequality. The reported difference in inequality between 2004(EI = − 0.090, *p* < 0.01) and 2017–18(EI = -0.87, *p* < 0.10) was marginal. However, significant reduction in horizontal inequity from 2004(HI = − 0.103, *p* < 0.01) to 2017–18(HI = − 0.048, *p* < 0.01) was estimated, indicating the convergence of inequity gap in treatment seeking over the years.

**Results: Inter-State Comparison**

In more developed states like Andhra Pradesh (EI = 0.07, 0.15 and 0.10), Kerala (EI = 0.10, 0.09 and 0.13) and West- Bengal (EI = 0.09,0.11 and 0.11), self-reported morbidity in entire study period of 2004, 2014 and 2017–18 was more concentrated amongst richer individuals as compared to all India average. Contrarily, North-Eastern states demonstrated small measures of inequality (EI = 0.01 and − 0.01) in 2004 and 2014 and perfect equality (EI = 0.00) in 2017–18.

Inter-state comparison of untreated morbidity underscored that inequality was concentrated amongst the poor in majority of Indian states, entailing that poor were more likely to leave the ailment untreated. The inter-state variation is notably elucidated in Figs. 5 and 6. However, it was transposed from pro-poor to pro-rich in some states such as Jammu and Kashmir, Haryana, West Bengal, Jharkhand, Madhya Pradesh and Kerala from 2004 to 2017–18.

Subsequently, perfect horizontal equity was achieved in Telangana in 2017–18(HI = 0.00).

**RESULTS: Decomposition**

A negative (positive) absolute contribution indicates that, if inequality in the outcome variable was determined by that correlate alone, then it would favour the better off (worse-off) and relative contributions (exhibiting how much percentage of inequality in the outcome measure is attributable to the inequality in contributing factor. Relative contribution is computed by dividing its absolute contribution by total inequality of outcome variable and multiplying it by 100) of each determinant and represented in Fig. 7 plotting the aggregate relative contributions of covariates in driving inequality. The descriptive statistics of all the covariates used in the study are elucidated in Table 3.

Overall, for self-reported morbidity, the relative contribution of need variables was about 21.4, 23 and 16.6% of unstandardized indices and the largest contribution to inequality stemmed from illegitimate factors accounting for 78, 77 and 83% of inequality in 2017–18, 2014 and 2004 respectively. The positive values for legitimate factors indicated that if self-reported morbidity were determined by need alone, it would be pro-rich.

Estimated coefficients of linear probability model exhibited that individuals belonging to backward and disadvantaged groups, residing in rural area, not covered under any health insurance scheme, residing in less developed state, having more than 10 members in the household and association with lower income/expenditure quintile groups were less likely to report morbidity.

The contribution of expenditure declined in generating the inequality in 2017–18, where, level of development of state was the biggest contributor amongst the factors amenable to policy intervention.

Whereas, being a male in 0–14 age group and suffering for more than 10 days from the illness significantly reduced the likelihood of having untreated morbidity in all the years.

**DISCUSSION AND CONCLUSIONS**

Our results also highlighted the role of having residence in rural area and absence of insurance cover in driving inequities which can also arise due to organizational barriers in detecting the morbidities. As the measure of morbidity is self-reported, it is sensitive to internal frame of reference and response styles. Hence, reporting heterogeneity can arise due to Positional Effect (Response shift) or Dispositional Effect/Judgement Effect. Heterogeneity in reporting due to attenuation bias arising from measurement error can be minimized with the help of Anchoring Vignettes using hypothetical stories or description of health problems which can then be adjusted and corroborated with individual’s subjective assessment of own situation [56]. Additionally, a multiple question instrument based on disease symptoms is recommended to reduce biases.

These states are also identified to be at advanced stages of epidemiological transition level (which is defined on the basis of ratio of Disability-Adjusted Life Years (DALYs), computed as the sum of years of potential life lost due to premature mortality and the years of productive life lost due to disability) from communicable disease to those from non-communicable disease and injuries combined) with burden of disease disproportionately skewed towards non-communicable diseases such as heart disease, diabetes, respiratory problems and cancer [57, 58]. Literature has established that reporting of morbidity is lower for non-communicable and chronic diseases vis. a vis. other disease conditions amongst poorer sections as ignoring minor symptoms/early signs of chronic diseases and detection bias is stronger for Non-Communicable Diseases (NCD’s) [21, 58].

To encapsulate the argument, it is understood that for most of the NCD conditions, diagnosis at health facility is the basis for the knowledge of presence of condition. This self-reporting, aided by facility-based diagnosis may transmute to an underestimation amongst poor because of their relatively low uptake and utilization of health services and greater likelihood of suffering from undiagnosed illness.

Untreated morbidity in all three study years was estimated to have pro-poor inequality and inequity as mirrored in negative values of Erreyger’s concentration index and Horizontal Inequity index. Although, there were favourable changes in un-treated morbidity between three time periods, there still remained considerable inequities that were disadvantageous to the poor. The results are congruous with other studies in Indian setting where distribution of sampled untreated ailing person was pro-poor [16, 61].

The reasons cited by the individuals for not seeking treatment were multifold that can be understood using Penchansky’s framework of access and utilization which is embedded in five dimensions i.e. Availability, Accessibility, Affordability, Acceptability and Accommodation [62]. In 2004, affordability (29.62%) and spatial accessibility which is interaction of accessibility and availability (10.56%) constituted important barriers to seek treatment. Albeit, health seeking behavior was mostly influenced by the reason of not considering the ailment serious (39.56%).

There are few caveats emanating from nature of data in this study and results should be interpreted with caution. Firstly, this dataset doesn’t circumscribe any objective measure of health/vignette schedule making it difficult to gauge the relative contribution of actual increase in disease burden and subjective perception bias in the levels of reported illness. Secondly, only individual and household level determinants were incorporated to explain self-reported morbidity and untreated morbidity, whereas, other factors pertaining to health system reforms, culture and behavior is not in the preview of this study due to data constraints. Thirdly, the information on outcomes and determinants was collected concurrently due to cross-sectional design, thus, associations rather than causal relationships are defined in the study.
