# Supplementary material for: Horizontal inequity in self-reported morbidity and untreated morbidity in India: Evidence from National Sample Survey Data
Source: Int J Equity Health. 2021 Jan 28;20:49. doi: 10.1186/s12939-020-01376-0 (PMC7842052; doi:10.1186/s12939-020-01376-0)
Supplement: Supplementary file 1 — Additional file 1. [file 12939_2020_1376_MOESM1_ESM.docx]

| **** | | | | | | | | | | | | | |
| --- | --- | --- | --- | --- | --- | --- | --- | --- | --- | --- | --- | --- | --- |
| **DOMINANCE TEST BETWEEN CONCENTRATION CURVE AND 45 DEGREE LINE** | | | | | | | | | | | | | |
| **Variable** | | **Significance** | | | | | | **Points** | | **Rule** | | **Decision** | |
| Self-reported morbidity | | 5% | | | | | | 19 | | MCA and IUP | | 45 degree dominates | |
| Self-reported morbidity(Std) | | 5% | | | | | | 19 | | MCA and IUP | | 45 degree dominates | |
| **** | | | | | | | | | | | | | |
| **Dominance Test between Generalized Curve and 45 degree Line** | | | | | | | | | | | | | |
| **Variable** | | **Significance** | | | **Points** | | | | **Rule** | | | **Decision** | |
| Self-reported morbidity | | 5% | | | 19 | | | | MCA and IUP | | | 45 degree dominates | |
| Self-reported morbidity(Std) | | 5% | | | 19 | | | | MCA and IUP | | | 45 degree dominates | |
| **** | | | | | | | | | | | | | |
| **Dominance Test between Standardized and Unstandardized Concentration Curves** | | | | | | | | | | | | | |
| **Variable** | | **Significance** | | | **Points** | | | | **Rule** | | | **Decision** | |
| Self-reported morbidity | | 5% | | | 19 | | | | MCA and IUP | | | Standardized dominates | |
|  | | | | | | | | | | | | | |
| **DOMINANCE TEST BETWEEN CONCENTRATION CURVE AND 45 DEGREE LINE** | | | | | | | | | | | | | |
| **Variable** | | | **Significance** | | | | **Points** | | | **Rule** | | **Decision** | |
| Self-reported morbidity | | | 5% | | | | 19 | | | MCA and IUP | | 45 degree dominates | |
| Self-reported morbidity(Std) | | | 5% | | | | 19 | | | MCA and IUP | | 45 degree dominates | |
|  | | | | | | | | | | | | | |
| **Dominance Test between Generalized Curve and 45 degree Line** | | | | | | | | | | | | | |
| **Variable** | | **Significance** | | | **Points** | | | | **Rule** | | | | **Decision** |
| Self-reported morbidity | | 5% | | | 19 | | | | MCA and IUP | | | | 45 degree dominates |
| Self-reported morbidity(Std) | | 5% | | | 19 | | | | MCA and IUP | | | | 45 degree dominates |
|  | | | | | | | | | | | | | |
| **Dominance Test between Standardized and Unstandardized Concentration Curves** | | | | | | | | | | | | | |
| **Variable** | | **Significance** | | | **Points** | | | | **Rule** | | **Decision** | | |
| Self-reported morbidity | | 5% | | | 19 | | | | MCA and IUP | | Standardized dominates | | |
|  | | | | | | | | | | | | | |
| **DOMINANCE TEST BETWEEN CONCENTRATION CURVE AND 45 DEGREE LINE** | | | | | | | | | | | | | |
| **Variable** | | **Significance** | | | **Points** | | | | **Rule** | | | **Decision** | |
| Self-reported morbidity | | 5% | | | 19 | | | | MCA and IUP | | | 45 degree dominates | |
| Self-reported morbidity(Std) | | 5% | | | 19 | | | | MCA and IUP | | | 45 degree dominates | |
|  | | | | | | | | | | | | | |
| **Dominance Test between Generalized Curve and 45 degree Line** | | | | | | | | | | | | | |
| **Variable** | | **Significance** | | | **Points** | | | | **Rule** | | | **Decision** | |
| Self-reported morbidity | | 5% | | | 19 | | | | MCA and IUP | | | 45 degree dominates | |
| Self-reported morbidity(Std) | | 5% | | | 19 | | | | MCA and IUP | | | 45 degree dominates | |
|  | | | | | | | | | | | | | |
| **Dominance Test between Standardized and Unstandardized Concentration Curves** | | | | | | | | | | | | | |
| **Variable** | | **Significance** | | | **Points** | | | | **Rule** | | **Decision** | | |
| Self-reported morbidity | | 5% | | | 19 | | | | MCA and IUP | | Standardized dominates | | |
|  | | | | | | | | | | | | | |
| **DOMINANCE TEST BETWEEN CONCENTRATION CURVE AND 45 DEGREE LINE** | | | | | | | | | | | | | |
| **Variable** | | **Significance** | | | **Points** | | | | **Rule** | | **Decision** | | |
| Untreated morbidity | | 5% | | | 19 | | | | MCA and IUP | | Concentration curve dominates | | |
| Untreated morbidity(Std) | | 5% | | | 19 | | | | MCA and IUP | | Concentration curve dominates | | |
|  | | | | | | | | | | | | | |
| **Dominance Test between Generalized Curve and 45 degree Line** | | | | | | | | | | | | | |
| **Variable** | | **Significance** | | | **Points** | | | | **Rule** | | **Decision** | | |
| Untreated morbidity | | 5% | | | 19 | | | | MCA and IUP | | 45 degree dominates | | |
| Untreated morbidity(Std) | | 5% | | | 19 | | | | MCA and IUP | | 45 degree dominates | | |
|  | | | | | | | | | | | | | |
| **Dominance Test between Standardized and Unstandardized Concentration Curves** | | | | | | | | | | | | | |
| **Variable** | | **Significance** | | | | **Points** | | | **Rule** | | | **Decision** | |
| Self-reported morbidity | | 5% | | | | 19 | | | MCA and IUP | | | Unstandardized dominates | |
|  | | | | | | | | | | | | | |
| **DOMINANCE TEST BETWEEN CONCENTRATION CURVE AND 45 DEGREE LINE** | | | | | | | | | | | | | |
| **Variable** | **Significance** | | | **Points** | | | | | **Rule** | | **Decision** | | |
| Untreated morbidity | 5% | | | 19 | | | | | MCA and IUP | | Concentration curve dominates | | |
| Untreated morbidity(Std) | 5% | | | 19 | | | | | MCA and IUP | | Concentration curve dominates | | |
|  | | | | | | | | | | | | | |
| **Dominance Test between Generalized Curve and 45 degree Line** | | | | | | | | | | | | | |
| **Variable** | | **Significance** | | **Points** | | | | | **Rule** | | **Decision** | | |
| Untreated morbidity | | 5% | | 19 | | | | | MCA and IUP | | Generalized curve dominates | | |
| Untreated morbidity(Std) | | 5% | | 19 | | | | | MCA and IUP | | Generalized curve dominates | | |
|  | | | | | | | | | | | | | |
| **Dominance Test between Standardized and Unstandardized Concentration Curves** | | | | | | | | | | | | | |
| **Variable** | | **Significance** | | **Points** | | | | | **Rule** | | **Decision** | | |
| Self-reported morbidity | | 5% | | 19 | | | | | MCA and IUP | | Standardized dominates | | |
|  | | | | | | | | | | | | | |
| **DOMINANCE TEST BETWEEN CONCENTRATION CURVE AND 45 DEGREE LINE** | | | | | | | | | | | | | |
| **Variable** | | **Significance** | | | **Points** | | | | **Rule** | | **Decision** | | |
| Untreated morbidity | | 5% | | | 19 | | | | MCA and IUP | | Concentration curve dominates | | |
| Untreated morbidity (Std) | | 5% | | | 19 | | | | MCA and IUP | | Concentration curve dominates | | |
|  | | | | | | | | | | | | | |
| **Dominance Test between Generalized Curve and 45 degree Line** | | | | | | | | | | | | | |
| **Variable** | | **Significance** | | | **Points** | | | | **Rule** | | **Decision** | | |
| Untreated morbidity | | 5% | | | 19 | | | | MCA and IUP | | Generalized curve dominates | | |
| Untreated morbidity (Std) | | 5% | | | 19 | | | | MCA and IUP | | Generalized curve dominates | | |
|  | | | | | | | | | | | | | |
| **Dominance Test between Standardized and Unstandardized Concentration Curves** | | | | | | | | | | | | | |
| **Variable** | | **Significance** | | | **Points** | | | | **Rule** | | **Decision** | | |
| Untreated morbidity | | 5% | | | 19 | | | | MCA and IUP | | Standardized dominates | | |
